# Relationships between Isomeric Metabolism and Regioselective Toxicity of Hydroxychrysenes in Embryos of Japanese Medaka (*Oryzias latipes*)

**DOI:** 10.1021/acs.est.2c06774

**Published:** 2022-12-27

**Authors:** Philip Tanabe, Daniela M. Pampanin, Hiwot M. Tiruye, Kåre B. Jørgensen, Rachel I. Hammond, Rama S. Gadepalli, John M. Rimoldi, Daniel Schlenk

**Affiliations:** † Environmental Toxicology Graduate Program, University of California, Riverside, California92521, United States; ‡ Department of Environmental Sciences, University of California, Riverside, California92521, United States; § Department of Chemistry, Bioscience and Environmental Engineering, University of Stavanger, Stavanger4021, Norway; ∥ Department of Biomolecular Sciences, The University of Mississippi School of Pharmacy, The University of Mississippi, University, Mississippi38677, United States; ⊥ Department of Chemistry, University of Illinois at Urbana-Champaign, Urbana, Illinois61801, United States

**Keywords:** hydroxychrysene, polycyclic aromatic hydrocarbons, developmental toxicity, oxy-PAHs, Japanese medaka, oil spills, cytochrome P450

## Abstract

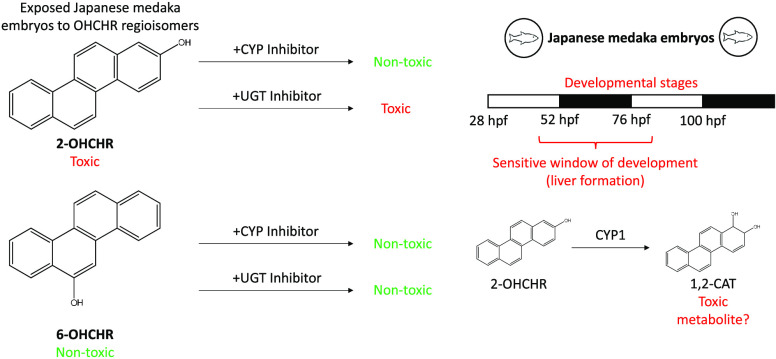

Oxygenated polycyclic aromatic hydrocarbons (oxy-PAHs) are ubiquitous contaminants that can be formed through oxidation of parent PAHs. Our previous studies found 2-hydroxychrysene (2-OHCHR) to be significantly more toxic to Japanese medaka embryos than 6-hydroxychrysene (6-OHCHR), an example of regioselective toxicity. We have also previously identified a sensitive developmental window to 2-OHCHR toxicity that closely coincided with liver development, leading us to hypothesize that differences in metabolism may play a role in the regioselective toxicity. To test this hypothesis, Japanese medaka embryos were treated with each isomer for 24 h during liver development (52–76 hpf). Although 6-OHCHR was absorbed 97.2 ± 0.18% faster than 2-OHCHR, it was eliminated 57.7 ± 0.36% faster as a glucuronide conjugate. Pretreatment with cytochrome P450 inhibitor, ketoconazole, reduced anemia by 96.8 ± 3.19% and mortality by 95.2 ± 4.76% in 2-OHCHR treatments. Formation of chrysene-1,2-diol (1,2-CAT) was also reduced by 64.4 ± 2.14% by ketoconazole pretreatment. While pretreatment with UDP-glucuronosyltransferase inhibitor, nilotinib, reduced glucuronidation of 2-OHCHR by 52.4 ± 2.55% and of 6-OHCHR by 63.7 ± 3.19%, it did not alter toxicity for either compound. These results indicate that CYP-mediated activation, potentially to 1,2-CAT, may explain the isomeric differences in developmental toxicity of 2-OHCHR.

## Abstract 2

Isomeric differences in developmental toxicity of hydroxychrysenes may be explained by differences in CYP450-mediated activation, potentially to catechol metabolites.

## 1 Introduction

Oxygenated polycyclic aromatic hydrocarbons (oxy-PAHs) are ubiquitous contaminants that can be formed through the photochemical or biological oxidation of parent PAHs.^1−3^ PAHs enter aquatic environments through runoff, atmospheric deposition, accidental discharge, and oil spills.^4−6^ While few environmental measurements of oxy-PAHs exist, they have been found at concentrations that are similar to or even higher than parent PAHs.^7,8^ Some groups have suggested that oxy-PAHs are “dead-end” products that will likely not break down any further, which could lead to accumulation as a legacy contaminant.^9−11^ The polar properties of oxy-PAHs increase their mobility within the environment that increases the risk of exposure to fauna and flora compared to parent PAHs.^12,13^ While analytical technologies have improved dramatically over the past few decades, it is still not feasible to measure every oxy-PAH in the environment. Therefore, narrowing the range of oxy-PAHs to monitor to specific compounds would be beneficial for regulators.

In addition to the potential for enhanced exposure, enhanced lethality, endocrine disruption, and developmental effects such as circulatory defects and malformations of the brain, jaw, and eyes have also been shown to be greater in exposures to oxy-PAHs relative to parent PAHs.^14,15^ Given the potential for enhanced formation during remediation events due to increased microbial oxidation,^16,17^ it is critical to better characterize the toxicity of oxy-PAHs in various environmental media.

Oxy-PAHs have been identified as quinones, carboxylic acids, and hydroxylated compounds with moieties at one or more carbon atoms within the molecule. Interestingly, regioselective toxicity has been observed for multiple isomers of several oxy-PAHs, making it difficult to predict adverse effects.^14,18,19^ For example, in zebrafish (*Danio rerio*), 2-hydroxychrysene (2-OHCHR) caused greater anemia in embryos than 6-hydroxychrysene (6-OHCHR), although 6-OHCHR was more lethal.^18^ However, in Japanese medaka (*Oryzias latipes*), 2-OHCHR caused both anemia and mortality, but 6-OHCHR caused neither, implying potential for species-specific effects.^19^ In addition, the parent compound chrysene did not cause any toxicity in either species.^18,19^

The toxicity of 2-OHCHR in Japanese medaka embryos closely coincided with liver development.^19^ Since the liver contains high concentrations of metabolic enzymes such as cytochrome P450s (CYPs), UDP-glucuronosyltransferases (UGTs), and sulfotransferases (SULTs),^20−22^ differences in metabolism between the isomers may play a role in the regioselective toxicities of 2- and 6-OHCHR in Japanese medaka. Hydroxy-PAHs can be detoxified through conjugation by UGTs and SULTs leading to metabolites that may be readily excreted from the body. Conversely, activation of hydroxylated PAHs may occur through subsequent hydroxylation of the phenolic compounds by CYP to a catechol, which could undergo further oxidation to a quinone, allowing the formation of semiquinone radicals through one-electron reduction reactions, which may generate reactive oxygen species (ROS). In excess concentrations, ROS deplete endogenous antioxidants and cause oxidative stress, potentially leading to damaged proteins, lipids, and DNA.^23,24^

Therefore, to explore the role of metabolism in the toxicity of 2-OHCHR, we investigated the following hypotheses: (1) 2-OHCHR is taken up more rapidly and has a longer embryonic half-life than 6-OHCHR, (2) 2-OHCHR undergoes phase I metabolism and activation at a greater rate than 6-OHCHR by CYP, (3) quinone metabolites of 2-OHCHR cause greater toxicity than 2-OHCHR or quinone metabolites of 6-OHCHR, and (4) 6-OHCHR undergoes conjugation and detoxification at a greater rate than 2-OHCHR.

## 2 Materials and Methods

### 2.1 Chemicals

2-OHCHR (>99% purity, Toronto Research Chemicals, Ontario, Canada) and 6-OHCHR (>99% purity, MRIGlobal, Kansas City, MO) were dissolved in DMSO and stored at −20 °C in a dark environment. Benzo(a)pyrene-3,6-quinone (BPQ) (>99% purity, Toronto Research Chemicals, Ontario, Canada) was dissolved in methanol and stored at −20 °C in a dark environment. Exposure solutions were made by dilution of stock solutions to 0.1% DMSO in deionized (DI) water. β-Glucuronidase from *Escherichia coli* (140 U/mg, Millipore-Sigma, St. Louis, MO) and sulfatase from *Helix pomatia* (200 units/mL, Millipore-Sigma, St. Louis, MO) were stored at 4 °C in a dark environment. 1,2- and 5,6-Quinones of chrysene (CHQ) were synthesized photochemically as described in Tiruye and Jørgensen,^25^ while 1,2-catechol (CAT) of chrysene was synthesized by the reduction of 1,2-quinone using NaBH_4_ based on the methods of Platt and Oesch^26^ and by photochemical cyclization of the corresponding 1,2-dimethoxy stilbene (detailed description in the Supporting Information). Both methods gave 1,2-CAT, fully characterized by nuclear magnetic resonance (NMR) and high-resolution mass spectrometry (HRMS) analyses. Synthesis of 2,8-dihydroxychrysene (DHC) was performed following a combination of previously published methods^27,28^ with modifications (detailed description in the Supporting Information). All analytes were identified by fluorescence absorption and mass spectral analyses (see the Supporting Information for details). A table of chemical names, CAS, LogKow, and structures is also provided in the Supporting Information.

### 2.2 Maintenance of Medaka Culture

Adult Japanese medaka were maintained under the UCR Institutional Animal Care and Use Committee (IACUC)-approved protocol (AUP#20190017). Collection of embryos occurred 1 h after the lights turned on. Embryos were evaluated for viability under a transmitted light microscope and sorted for the correct stage (<4 hpf) following staging guidelines from Iwamatsu et al.^29^ for stage-specific morphological characteristics. Japanese medaka were maintained at 28 °C on a 14 h:10 h light:dark cycle.

### 2.3 Exposure Regime

Thirty embryos at 4 hpf were selected from a previously sorted pool and placed in glass petri dishes (60 × 15 mm) for exposure. Three replicate petri dishes were utilized, each containing 30 embryos. Exposure solutions were made fresh daily by dilution of stock solutions to 0.1% DMSO at appropriate nominal concentrations. The embryos were exposed to 0.5, 3, and 5 μM 1,2-CHQ or 5,6-CHQ from 4 to 172 hpf to test the toxicities of these putative hydroxychrysene metabolites. A vehicle control of 0.1% DMSO water underwent the same exposure regime. The concentrations of OHCHRs and CHQs for treatments were based upon a previous study in our laboratory.^19^

Ten embryos at 52 hpf were exposed to 5 μM 2- or 6-OHCHR for uptake and depuration analysis. Embryos were exposed for 2, 4, 8, 12, and 24 h for uptake exposures. For depuration exposures, embryos were exposed for 24 h, washed with deionized (DI) water, then transferred to 0.1% DMSO water in a clean petri dish to allow depuration. Depuration exposures lasted for 4, 8, 12, and 24 h following transfer to water. As mentioned above, the duration and timing of exposures were based on our earlier studies and the timing of liver formation within Japanese medaka.^19^

To explore the role of cytochrome P450s in the toxicity of 2-OHCHR, 30 embryos were exposed to 20 μM ketoconazole, a broad-spectrum cytochrome P450 inhibitor, from 28 to 52 hpf then to 10 μM 2-OHCHR from 52 to 76 hpf. Embryos were also pretreated with 10 μM nilotinib, a UGT inhibitor, to assess the effects of glucuronidation on hydroxychrysene toxicity. Embryos (30 pooled individuals) were then transferred to 0.1% DMSO water until 172 hpf. Embryos were washed prior to transfer to new exposure solutions and a new petri dish was used for each exposure to minimize retention of residual chemicals. Ethoxyresorufin-*O*-deethylase (EROD) activity and measurements of hydroxylated parent compounds within embryos were used to determine the respective efficacy of the inhibitors. To determine if pretreatments with inhibitors provided protection or exacerbated toxicity, anemia and daily mortality were evaluated under transmitted light using an Accu-Scope 3000 microscope as previously described.^19^ Anemia was defined by no visible blood cells and mortality was identified by no observable heartbeat.

### 2.4 EROD Imaging

EROD activity was measured by following methods described in Nacci et al.^30^ with slight modifications. Embryos were exposed to 10 μM 2- or 6-OHCHR and 21 μg/mL 7-ethoxyresorufin (ER) from 52 to 76 hpf. Following exposures, embryos were anesthetized with MS-222, washed with DI water, and then transferred to a petri dish with molded agarose for imaging. Resorufin was quantified within embryos using fluorescence microscopy. A Keyence BZ-X710 microscope was used. Embryos were positioned in molded agarose dorsally with the heart centered within the image. Image analysis was conducted in ImageJ version 1.8.0. The circumferences of the embryos were traced, and the integrated density values of the selection were measured for each embryo. High integrated density values corresponded to high EROD activity.

### 2.5 Sample Preparation

Following exposure from 52 to 76 hpf, Japanese medaka embryos were washed with DI water three times. The water was then removed, and the embryos were dried and weighed before being transferred to a clean 1.5 mL polypropylene conical centrifuge tube. The embryos were flash-frozen in liquid nitrogen and then stored at −80 °C in a dark environment until extraction.

To measure the metabolism of OHCHRs in embryos, treatment samples of 30 pooled embryos underwent solid phase extraction (SPE). To identify potential glucuronides or sulfates as putative metabolites, 200 μL of DI water was added to 1.5 mL centrifuge tubes and the embryos were homogenized with a pestle homogenizer for 1 min. Glucuronidase (100 U), sulfatase (10 U), or 50 μL of DI water was then added, and the vial was placed in a temperature-controlled shaker at 37 °C for 1 h. Methanol (200 μL) and 50 μL of 2 μg/mL BPQ were added as an internal standard to the tube and underwent further homogenization for 1 min. The homogenate was then transferred to a glass test tube with the subsequent addition of 500 μL of methanol and 4000 μL of DI water. The final concentration of the homogenate was 15% methanol and 85% DI water.

To enrich putative metabolites from embryonic treatments for chromatographic measurements, Waters Sep-Pak C18 SPE cartridges (3 cm^3^, 1 g sorbent, 55–105 μm particle size) were utilized for extractions on a vacuum manifold. Cartridges were conditioned with 5 mL of methanol and then equilibrated with 5 mL of 15% methanol in DI water. Samples were then loaded onto the cartridges and then washed with 15% methanol in DI water. Cartridges were then eluted with 10 mL of methanol into 20 mL glass scintillation vials. The eluents were blown down to dryness under a stream of nitrogen gas in a water bath at 40 °C. Samples were reconstituted in 500 μL of acetonitrile (ACN), vortexed for 10 s, and then transferred to autosampler vials for chromatographic analysis. Recovery values ranged from 79 to 97% based on the total loss of internal standard (BPQ) in each extract.

### 2.6 Chromatographic Conditions

A Shimadzu Prominence-i LC-2030 HPLC system with an RF-10AXL fluorometer was used for chromatographic analysis. Samples were injected onto a C18 column (4.6 × 150 mm, Shiseido, Japan) at a flow rate of 1 mL/min. The column temperature was maintained at 40 °C. All mobile phases were acidified with formic acid at a concentration of 0.1% by volume. Initial conditions were 30% ACN/H_2_O. The chromatographic conditions were as follows: gradient to 60% ACN/H_2_O from 0 to 25 min, gradient to 95% ACN/H_2_O from 25 to 27 min, maintain 95% ACN/H_2_O from 27 to 29 min, gradient to 30% ACN/H_2_O from 29 to 30 min, and maintain 30% ACN/H_2_O from 30 to 34 min. Retention times of analytes were as follows: 2,8-DHC: 14.159, 1,2-CAT: 16.575, 5,6-CAT: 17.856, 2-OHCHR: 18.361, 6-OHCHR: 18.842, and chrysene: 20.336. Since quinones fluoresce poorly, UV/vis detection at 254 nm was required for their detection. Their retention times were as follows: 1,2-CHQ: 17.246, 5,6-CHQ: 17.704, and BPQ: 18.083. The limit of quantification was 10 ng/mL for all compounds.

### 2.7 Metabolism of 2- and 6-OHCHR

OHCHRs, CHQs, and CATs were identified based on coelution with analytical standards using ultraviolet (UV) absorbance or fluorescence, as well as fragmentation patterns using HRMS (Figures S1–S4). Glucuronide and sulfate conjugates were quantified by calculating the difference between parent OHCHR concentrations in extracts treated with glucuronidase or sulfatase and in extracts without deconjugation enzyme treatments.

Percent conversion and mass balance analyses of 2- and 6-OHCHR were conducted after 24 h uptake beginning at the 52 hpf timepoint, since liver formation begins to occur at this developmental stage. As noted above, each treatment contained 30 embryos at 52 hpf. Water concentrations after 24 h uptake were compared to initial water concentrations and the difference was assumed to have either entered the embryos or adsorbed to the glass petri dish or to the surface of embryos. To quantify OHCHRs adsorbed to the petri dishes, all water was removed from the dishes, the dishes were dried completely, and then washed with 10 mL of acetone and 10 mL of hexane. Acetone and hexane were collected in a glass scintillation vial, blown to dryness under a stream of nitrogen gas, then reconstituted in 1 mL of ACN and transferred to a 2 mL screw thread vial for liquid chromatography (LC) analysis. OHCHRs adsorbed to the surface of embryos that were removed during the wash step were quantified by collecting the water from the embryo washes that underwent SPE extraction using the same protocol described above. The OHCHRs in the embryos were further divided into parent forms and metabolites, consisting of glucuronides, sulfates, CATs, and CHQs based on coelution with standards.

### 2.8 Uptake and Elimination Rate Constants

Concentrations of 2- and 6-OHCHR were calculated using body burden values from glucuronidase and sulfatase-treated extracts. Due to glucuronides being the major metabolite after 24 h uptake, additional timepoints were sampled for glucuronide metabolite concentrations. This allowed the calculation of absorption (*K*_ab_) and elimination (*K*_el_) rate constants for 2- and 6-OHCHR using body burden values from glucuronidase-treated extracts by linear regression analysis.

### 2.9 Statistical Analysis

All statistical analyses were conducted in Rstudio version 1.2.5019 and IBM SPSS Statistics 27. All data were checked for normality and equal variance assumptions by plotting residuals and quantiles of the data sets. Rate constants and body burdens between 2- and 6-OHCHR were compared using two-sample *t*-tests. Percent mortality and percent anemic phenotype data were compared to the control using a generalized linear model. EROD activities were compared using a two-way ANOVA and a post hoc Tukey HSD test. EROD comparisons utilized 30 replicates, while the rest of the statistical tests were conducted using three replicates. A *p*-value of 0.05 was used for all statistical tests. All figures were generated using GraphPad Prism version 8.4.3.

## 3 Results

### 3.1 Metabolism of 2- and 6-OHCHR

**Figure 1:**
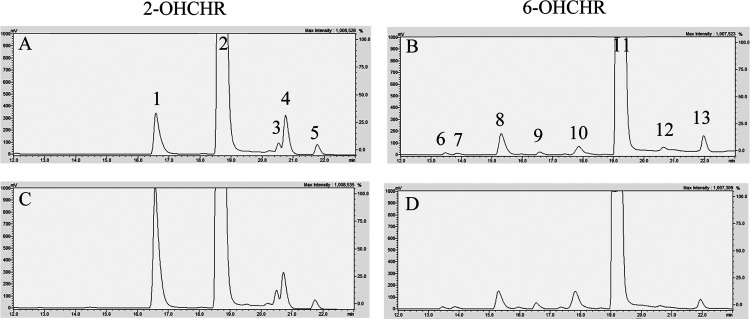
Representative chromatograms of tissue extracts incubated with glucuronidase and sulfatase following treatment with 2- or 6-hydroxychrysene (OHCHR) from 52 to 76 hpf. (A) and (B) were pretreated with 20 μM ketoconazole (CYP inhibitor) from 28 to 52 hpf, while (C) and (D) were not. Peaks 1 and 10 indicate a 1,2-catechol and 5,6-catechol metabolite, while peaks 2 and 11 indicate the parent OHCHR. All other peaks are unknown metabolites.

Approximately 4.22 ± 1.23% aqueous 2-OHCHR and 9.17 ± 1.03% aqueous 6-OHCHR were taken up by the embryos after 24 h (Table S1). Significant differences in the conversion of parent compounds to sulfates, CATs, and CHQs were observed between OHCHRs; 68.1 ± 24.1% more 2-OHCHR was converted to sulfate conjugated compared to 6-OHCHR and 370 ± 59.9% more 2-OHCHR was converted to a CAT compared to 6-OHCHR. While 18.0 ± 4.58% of 6-OHCHR was converted to 5,6-quinone, quinones were not detected in 2-OHCHR extracts (Table 1). Figure 1 is a representative chromatogram of an embryonic extract treated with OHCHRs. In addition to CATs and CHQs (for 6-OHCHR only), several other metabolites were observed. Based on retention times and mass spectra, these may have been hydroxy-dihydrodiols, triols, or methylated metabolites. To assess the possibility of 2,8-dihydroxychrysene formation, this putative metabolite was synthesized and ran as a standard following chrysene treatment in embryos. However, coelution was not observed. In addition, other putative compounds could not be quantified due to the limit of detection (LOD) for LC/MS and limited availability of analytical standards.

**Table 1:** Conversion of 2- and 6-Hydroxychrysene (OHCHR) Metabolites within Embryos after 24 h Uptake^a^

type of metabolite	2-OHCHR	6-OHCHR
% parent	10.8 ± 3.63	4.92 ± 2.26
% glucuronide	31.8 ± 2.92	34.6 ± 3.14
% sulfate*	25.1 ± 2.24	18.7 ± 2.01
% catechol*	20.9 ± 1.68	4.64 ± 1.34
% quinone*	BLD	18.0 ± 4.58
% unknown	11.49 ± 2.22	19.1 ± 7.39

a Percent parent represents unmetabolized OHCHRs and percent unknown represents uncharacterized metabolites. Asterisk represents a significant difference between 2- and 6-OHCHR revealed by a *t*-test. Values represent the mean of three replicates ± SEM.

### 3.2 Toxicokinetics of 2- and 6-OHCHR

**Figure 2:**
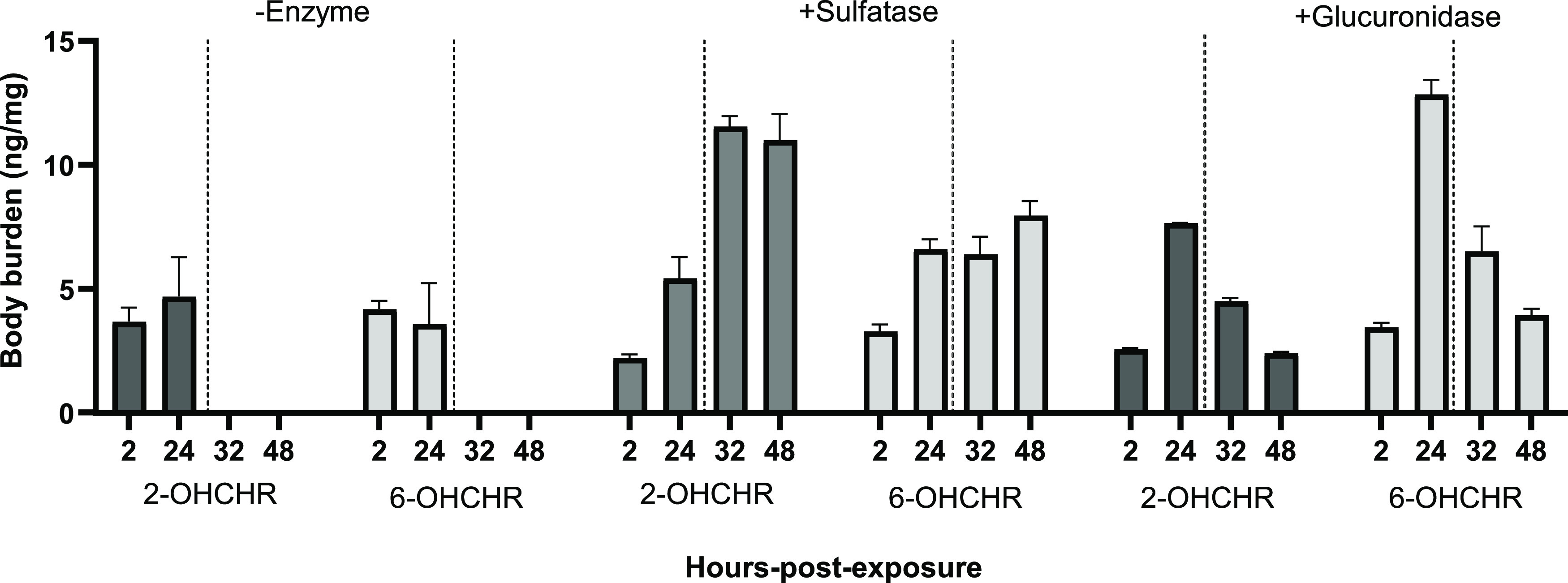
Body burdens of hydroxychrysenes (OHCHRs) in Japanese medaka embryos after 2 or 24 h of uptake and 12 or 24 h of depuration. Dotted lines indicate the transfer of embryos from exposure solution to water to allow depuration (after 24 h exposure). Depuration body burdens are indicated by 32 and 48 that correspond to 12 and 24 h of depuration, respectively. Values represent the mean of three replicates. Error bars represent ±SEM.

**Figure 3:**
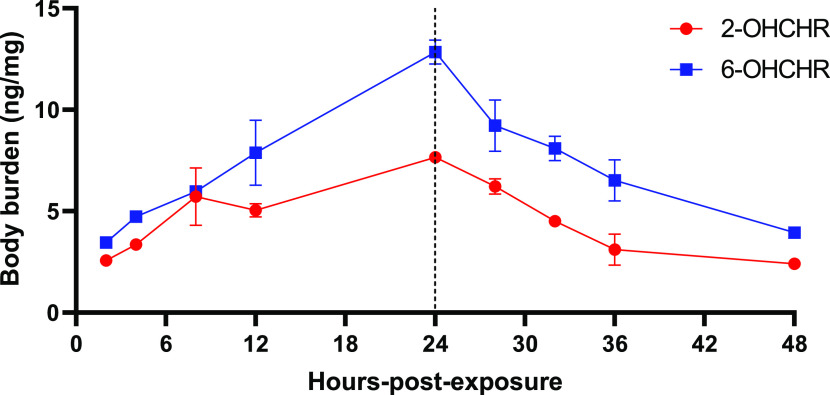
Uptake and depuration body burdens of 2- and 6-hydroxychrysene (OHCHR)-treated Japanese medaka embryos following treatment with glucuronidase. Dotted lines indicate the transfer of embryos from exposure solution to water to allow depuration (after 24 h exposure). Body burdens were measured after 2, 4, 8, 12, or 24 h of uptake. Depuration body burdens were determined at 28, 32, 36, and 48 h that correspond to 4, 8, 12, and 24 h of depuration. Values represent the mean of three replicates. Error bars represent ±SEM.

No significant differences in body burden were found at 2 or 24 h uptake between 2- and 6-OHCHR (Figure 2). All depuration body burden measurements for 2- or 6-OHCHR were below the LOD at these timepoints. However, when samples were treated with glucuronidase or sulfatase, the resulting hydroxylated compounds allowed the calculation of absorption and elimination rates for the metabolites. Significant differences were found between body burdens of extracts treated with and without glucuronidase or sulfatase at 24 h uptake, as well as during 12 and 24 h depuration (Figure 2). To better estimate rates of uptake and conjugation, glucuronides were measured at additional timepoints (Figure 3). Absorption rates for 2- and 6-OHCHR glucuronides were 2.14 ± 0.08 and 4.20 ± 0.29 ng/mg·h, respectively. Depuration rates for 2- and 6-OHCHR glucuronides were 2.16 ± 0.09 and 3.41 ± 0.35 ng/mg·h, respectively. Significant differences in both absorption and elimination rates were found between 2- and 6-OHCHR (*p* < 0.05) with 6-OHCHR being taken up and eliminated more rapidly than 2-OHCHR.

### 3.3 Toxicity and Uptake of 1,2- and 5,6-CHQ

Exposures to 1,2- or 5,6-CHQ did not result in significant anemia or mortality at any tested concentration (Tables S2 and S3). Body burdens of 1,2- and 5,6-CHQ within embryos following exposure to 3 μM for 24 h from 52 to 76 hpf were 17.9 ± 1.01 and 9.68 ± 0.77 nmol/mg, respectively (Figure S5).

### 3.4 CYP and UGT Inhibitor Pretreatments

**Figure 4:**
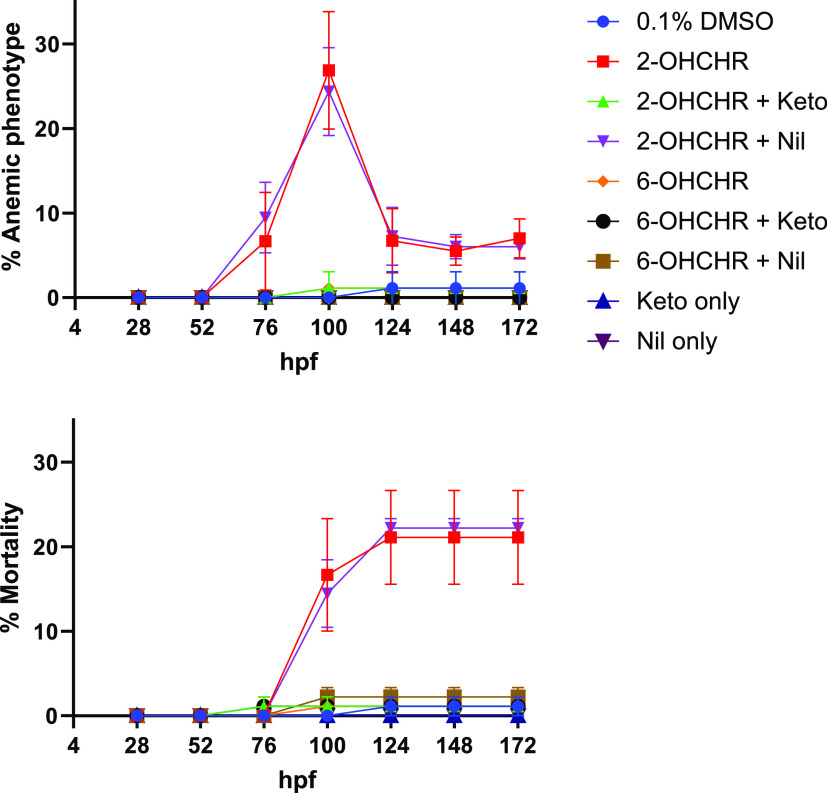
Percent anemic phenotype and mortality of medaka embryos pretreated with 20 μM ketoconazole (CYP inhibitor) or 10 μM nilotinib (UGT inhibitor) from 28 to 52 hpf, exposed to 10 μM 2- or 6-hydroxychrysene (OHCHR) from 52 to 76 hpf, and then transferred to 0.1% DMSO until 172 hpf. Values represent the mean of three replicates. Error bars represent ±SEM.

**Figure 5:**
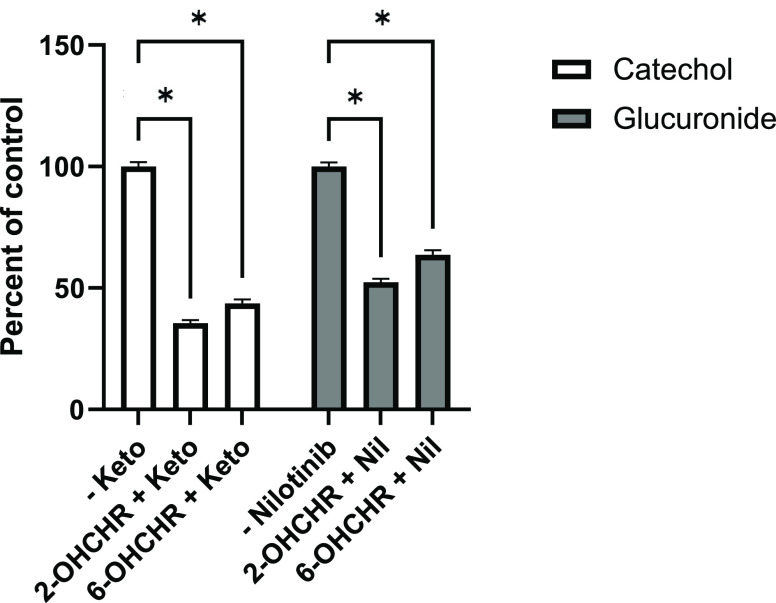
Concentrations of catechols and glucuronides of 2- and 6-OHCHR following pretreatments with the CYP inhibitor (ketoconazole) and UGT inhibitor (nilotinib) compared to controls (no inhibitor). Asterisks (*) represent significant differences in metabolite concentrations with and without the inhibitor. Values represent the mean of three replicates ±SEM.

**Figure 6:**
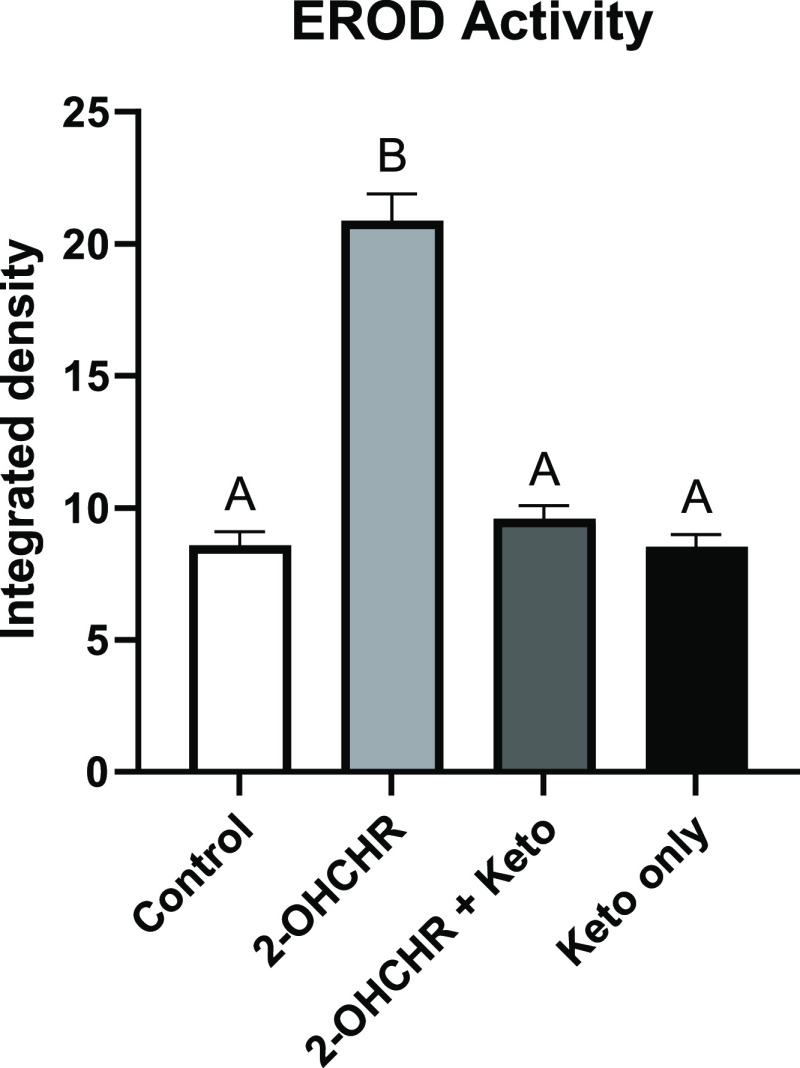
EROD activity following treatments with 2-hydroxychrysene (OHCHR) from 52 to 76 hpf, with or without ketoconazole (keto) pretreatment from 28 to 52 hpf. Letters indicate significant differences determined by a two-way ANOVA followed by a post hoc test. Values represent the mean of 30 replicates ±SEM.

Significant differences in percent mortality and anemia were found at 100 hpf between 10 μM 2-OHCHR treatments from 52 to 76 hpf and embryos that were pretreated with 20 μM ketoconazole from 28 to 52 hpf (Figure 4). Percent anemia and mortality were 96.8 ± 3.19 and 95.2 ± 4.76% lower in ketoconazole pretreatments compared to 2-OHCHR treatments, respectively. CYP inhibition caused a 64.4 ± 2.14% reduction of chrysene-1,2-diol (CAT), a phase I CYP metabolite of 2-OHCHR (Figure 5). Significant differences in EROD activity were observed between 10 μM 2-OHCHR-treated embryos with and without 20 μM ketoconazole pretreatments, with a 90.8 ± 7.00% reduction in EROD activity in pretreated individuals when normalized to controls (Figure 6). Pretreatments with 10 μM nilotinib did not affect toxicity of 10 μM 2- or 6-OHCHR treatments (Figure 4) but did significantly reduce glucuronide formation by 52.4 ± 2.55 and 63.7 ± 3.19% (Figure 5).

## 4 Discussion

Previous studies in our laboratory indicated isomeric differences in toxicity between 2- and 6-OHCHR in embryos of Japanese medaka, 2-OHCHR being more potent than 6-OHCHR.^18,19^ A sensitive window of development was identified between 52 and 100 hpf, which closely coincided with liver development. The liver contains high concentrations of metabolic enzymes, such as CYP, UGT, and SULT. Therefore, we hypothesized that metabolism may play a role in the regioselective toxicities of 2- and 6-OHCHR.

Conversion of 2- and 6-OHCHR to metabolites differed between compounds after 24 h of treatment at 52 hpf. While formation of glucuronides was similar, sulfates constituted a significantly higher proportion of total metabolites of 2-OHCHR than 6-OHCHR, although there was no significant difference in concentrations. Although no significant differences were found in proportions of glucuronide between 2- and 6-OHCHR, it was the major metabolite for both compounds at 24 h with significantly higher concentrations compared to controls. Due to it being the major metabolite, glucuronide formation was further analyzed for toxicokinetic parameters and their effects on OHCHR toxicities.

While uptake was greater with the less toxic 6-OHCHR, conversion and elimination to predominantly conjugated metabolites was also faster, indicating conjugation to metabolites may be a factor explaining the toxicity of either compound (Figure 1). Although the concentrations of both OHCHRs in glucuronidase-treated extracts decreased following depuration, the concentrations significantly increased in sulfatase-treated 2-OHCHR extracts and did not change in 6-OHCHR extracts. This indicates that sulfate conjugates may not be readily eliminated like glucuronide conjugates and instead may be retained within the embryos. However, due to the lack of available SULT inhibitors, the effects of inhibiting sulfation could not be assessed. Glucuronides are more hydrophilic compared to sulfates, which make them more likely to be eliminated from the body. Schebb et al.^31^ observed that in larval Japanese medaka treated with triclocarban, glucuronide was the major metabolite and was eliminated more rapidly compared to the sulfate conjugate. However, Van Wijk et al.^32^ observed that zebrafish larvae excreted sulfate conjugates of paracetamol more rapidly compared to glucuronides. It should be noted, however, that paracetamol sulfate was the major metabolite and was observed at a 10-fold higher concentration within the larvae compared to glucuronide. In a study with benzo(a)pyrene (BaP), Hornung et al.^33^ observed little to no elimination of metabolites from Japanese medaka embryos between 1 and 7 dpf but observed elimination post-hatch, contrasting our results. However, this study only quantified a single glucuronide metabolite (BaP-3-glucuronide) and sulfates were neither targeted for analysis nor detected, which could indicate that other phase II metabolites could have been missed. Additionally, since there are few toxicokinetic studies of PAHs, let alone oxy-PAHs, in fish embryos, the distribution of different PAHs or oxy-PAHs within embryos could vary, which may explain the differences observed with other studies.

Given the relatively high conversion of 2- and 6-OHCHR to glucuronides, inhibiting conjugation reactions could potentially enhance the toxicity of these compounds. Nilotinib is a potent UGT1A1 inhibitor, which has been characterized in mammalian models but not in fish.^34−36^ Significant reductions in overall glucuronides were observed in embryonic treatments, showing that this compound was effective as a UGT inhibitor in Japanese medaka embryos. However, no significant change in toxicity was observed, suggesting diminished glucuronidation of 2- or 6-OHCHR may not have significant influence on the regioselective differences in toxicity.

While conjugation may not be an important pathway of detoxification, sequential oxidation of hydroxy-PAHs to dihydroxy derivatives, catechols, or quinones has been shown to enhance the toxicity of phenolic PAHs.^14,37,38^ Exposures to putative quinone metabolites of 2- and 6-OHCHR did not result in significant anemia or mortality. Quinone metabolites were observed following treatment with 6-OHCHR in Japanese medaka embryos (5,6-CHQ), but quinones were not detected following treatments with the more toxic 2-OHCHR. Body burden measurements following 24 h treatment with both quinones were similar to concentrations of 2- and 6-OHCHR, indicating uptake occurred with embryonic concentrations approaching that of 2-OHCHR. Since similar concentrations of quinones and 2-OHCHR were observed within embryos, and toxicity was not observed with quinone treatments, quinone formation may not be a critical step in the isomeric differences in embryotoxicity. While 1,2-CHQ was neither detected nor toxic to embryos, other unidentified quinones may play more of a role. *para*-Quinones tend to be more toxic relative to *ortho*-quinones. Knecht et al.^14^ observed that while 9,10-anthraquinone was not toxic to zebrafish embryos, 1,4-anthraquinone was very toxic. However, the same study also observed similar levels of toxicity between *ortho* and *para*-quinones of phenanthrene and naphthalene, which suggests that the trend may be PAH-specific. *para*-Quinones also tend to be more stable than *ortho*-quinones,^39^ which may contribute to greater half-lives for *para*-quinones, allowing higher concentrations for potential redox cycling and the generation of ROS. Since several unknown metabolites were present in chromatograms from embryos treated with 2-OHCHR, further analyses were performed to generate para-metabolites of chrysene, such as the 2,8-dihydroxy metabolite. However, formation of this metabolite was not observed in embryos. Thus, additional spectroscopic analyses may be necessary to determine if other compounds may be more important.

One metabolite that was identified in 2-OHCHR treatments that correlated with the anemic phenotype and mortality was 1,2-CAT, a catechol of chrysene. Catechols are commonly formed via oxygenation of phenolic metabolites catalyzed by CYP.^40−43^ To determine if CYP played a role in 2-OHCHR toxicity, embryos were pretreated with ketoconazole, a broad-spectrum CYP inhibitor, prior to 2-OHCHR exposure. Formation of 1,2-CAT was significantly reduced with the CYP inhibitor and pretreatment provided significant protection from both 2-OHCHR-induced anemia and mortality. Previous in vitro studies also indicated that 2-OHCHR has a four-fold higher affinity to aryl hydrocarbon receptors compared to 6-OHCHR,^44,45^ suggesting 2-OHCHR may induce CYP1 orthologues at a greater rate than 6-OHCHR. Treatment of embryos with 2-OHCHR induced the CYP1 enzyme EROD activity and pretreatment with ketoconazole significantly diminished EROD activity (Figure 6), suggesting CYP1 may be involved in CAT formation and toxicity. Ketoconazole has also been shown to inhibit CYP1 and CYP3 catalytic activities in rainbow trout, Atlantic cod, and killifish.^21,46,47^

**Figure 7:**
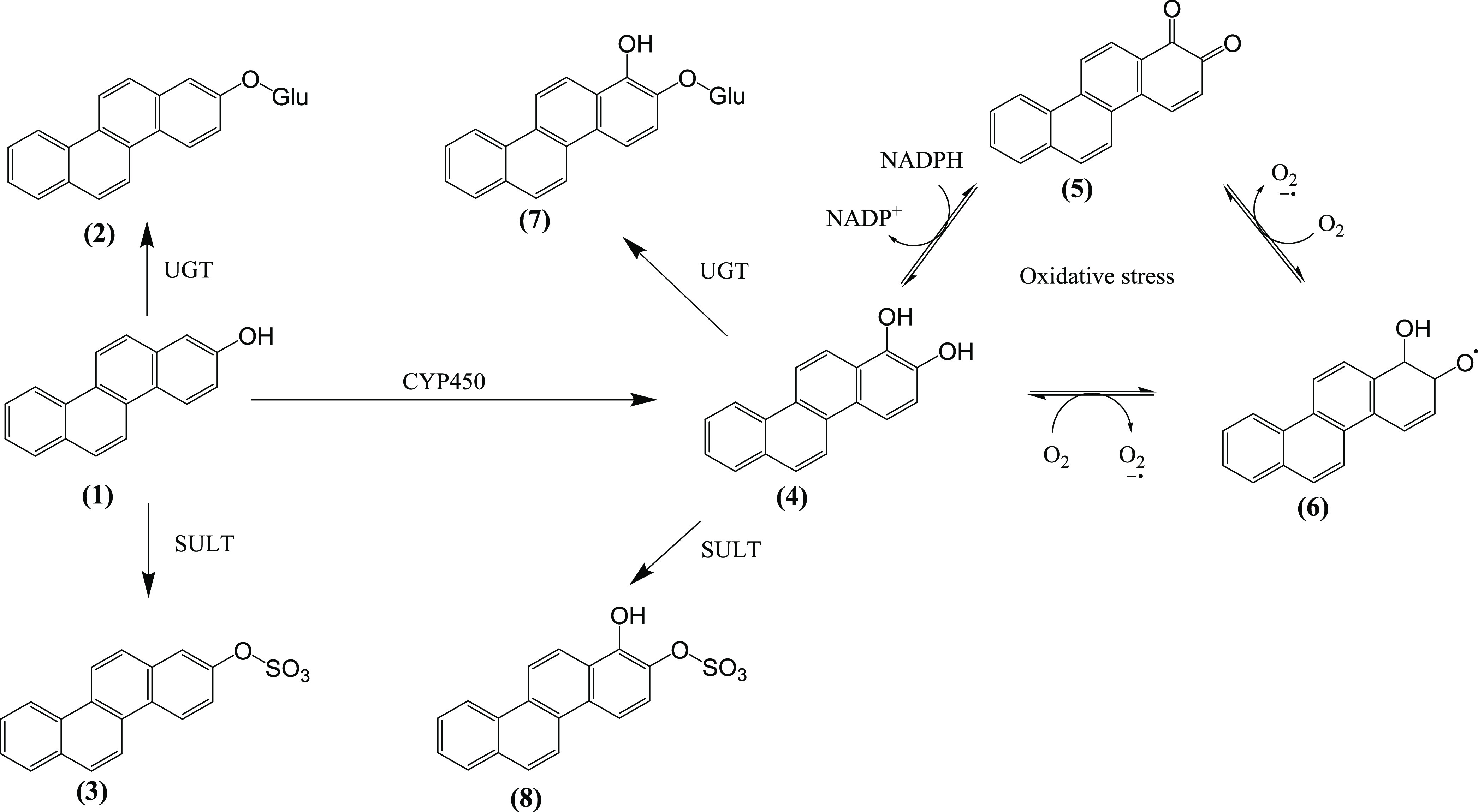
Theoretical metabolic pathway of hydroxy-polycyclic aromatic hydrocarbons using 2-OHCHR as a model compound: (1) 2-hydroxychrysene, (2) chrysene-2-O-glucuronide, (3) chrysene-2-sulfate, (4) chrysene-1,2-diol (catechol), (5) chrysene-1,2-quinone, (6) chrysene-1,2-semiquinone radical, (7) 1-hydroxychrysene-2-O-glucuronide, and (8) 1-hydroxychrysene-2-sulfate.

A hypothetical mechanism of action may involve oxidation of 1,2-CAT to a semiquinone radical, which may elicit oxidative stress (Figure 7). This has been observed in other studies with catechols of benzene, naphthalene, and benzo(a)pyrene where semiquinone radical formation from catechols resulted in toxicity by binding to macromolecules or through redox cycling.^48−52^ Further studies are needed to determine the mechanistic relevance of oxidative stress and its relationship to the anemic phenotype and/or mortality associated with 2-OHCHR.

In summary, metabolism likely plays a significant role in 2-OHCHR toxicity. While uptake and depuration as a glucuronide conjugate were significantly higher for 6-OHCHR than for 2-OHCHR, pretreatments with a UGT inhibitor did not affect the toxicity of either compound. However, pretreatments with a CYP inhibitor significantly reduced the toxicity of 2-OHCHR, indicating that a toxic metabolite may contribute to its toxicity. A significantly higher proportion of 2-OHCHR was converted to a catechol metabolite compared to 6-OHCHR, the formation of which was significantly reduced following pretreatments with a CYP inhibitor along with toxicity. These results indicate that 1,2-CAT may be a toxic metabolite, which plays a role in 2-OHCHR toxicity in embryonic Japanese medaka.

## Supporting Information Available

Part 1: analysis; mass balance of 2- and 6-OHCHR (Table S1); percent anemic phenotype in medaka embryos exposed to 1,2- and 5,6-CHQ (Table S2); percent mortality in medaka embryos exposed to 1,2- and 5,6-CHQ (Table S3); information for chemicals examined in this study (Table S4); high-resolution mass spectra (HRMS) of 2-OHCHR (Figure S1); HRMS of 6-OHCHR (Figure S2); HRMS of 5,6-CHQ (Figure S3); HRMS of 1,2-catechol (Figure S4); body burdens of 1,2- or 5,6-CHQ in medaka embryos (Figure S5); chromatograms of tissue extracts of embryos exposed from 52 to 76 hpf 2- or 6-OHCHR (Figure S6); fluorescence chromatograms of sodium borohydride reductions of 1,2- and 5,6-CHQ (Figure S7); sodium borohydride reduction method (Text S1); mass spectrometric identification (Text S2); Part 2: synthesis of 1,2-dihydroxychrysene (1,2-catechol); and Part 3: synthesis of 2,8-dihydroxychrysene (PDF)
