# Supplementary material for: Relationships between Isomeric Metabolism and Regioselective Toxicity of Hydroxychrysenes in Embryos of Japanese Medaka (Oryzias latipes)
Source: Environ Sci Technol. 2022 Dec 27;57(1):539–48. doi: 10.1021/acs.est.2c06774 (PMC9835889; doi:10.1021/acs.est.2c06774)
Supplement: Supplementary file 1 — es2c06774_si_001.pdf [file es2c06774_si_001.pdf]

Supplemental Information

Relationships between isomeric metabolism and regioselective toxicity of hydroxychrysenes in embryos of Japanese medaka *(Oryzias latipes)*

Philip Tanabe[1,2], Daniela M. Pampanin[3], Hiwot M. Tiruye[3], Kåre B. Jørgensen[3], Rachel I. Hammond[5], Rama S. Gadepalli[4], John M. Rimoldi[4], Daniel Schlenk[2]*

[1]Environmental Toxicology Graduate Program, University of California, Riverside, CA

[2]Department of Environmental Sciences, University of California, Riverside, CA

[3]Department of Chemistry, Bioscience and Environmental Engineering, University of Stavanger, Stavanger, Norway

[4]Department of Biomolecular Sciences, The University of Mississippi College of Pharmacy, The University of Mississippi, University, MS

[5]Department of Chemistry, University of Illinois at Urbana-Champaign, Urbana, IL

There are 29 pages with 7 Supplementary Figures and 4 Supplementary Tables.

** Corresponding author:*

Corresponding author: Philip Tanabe

Corresponding email: philip.tanabe@noaa.gov

Corresponding address: Hollings Marine Laboratory, National Oceanic and Atmospheric Administration, 331 Fort Johnson Rd, Charleston, SC 29412

Part 1: Analysis

Table S1. Mass balance of 2- and 6-OHCHR adsorbed to the exposure dish (glass) and onto embryos, taken up by the embryos, and how much remained within the water. Values represent the mean of three replicates ± SEM.

Location	2-OHCHR	6-OHCHR
% in water	76.5 ± 0.948	74.5 ± 0.921
% on glass	16.9 ± 1.03	11.5 ± 1.439
% on embryo	2.29 ± 1.317	4.76 ± 1.657
% uptake	4.22 ± 1.23	9.17 ± 1.03

Table S2. Percent anemic phenotype of medaka embryos exposed to 0.5, 3, or 5 µM 1,2- or 5,6-chrysenequinone (CHQ) from 52-76 hpf. Values represent the mean of three replicates. Error bars represent ± SEM.

	1,2-CHQ			5,6-CHQ		
Maturity	0.5 µM	3 µM	5 µM	0.5 µM	3 µM	5 µM
28 hpf	0 ± 0	0 ± 0	0 ± 0	0 ± 0	0 ± 0	0 ± 0
52 hpf	0 ± 0	0 ± 0	0 ± 0	0 ± 0	0 ± 0	0 ± 0
76 hpf	0 ± 0	0 ± 0	0 ± 0	0 ± 0	0 ± 0	0 ± 0
100 hpf	0 ± 0	0 ± 0	1.11 ± 1.11	0 ± 0	0 ± 0	0 ± 0
124 hpf	0 ± 0	0 ± 0	1.11 ± 1.11	0 ± 0	0 ± 0	1.67 ± 1.11
148 hpf	0 ± 0	0 ± 0	1.11 ± 1.11	0 ± 0	0 ± 0	1.67 ± 1.11
172 hpf	0 ± 0	0 ± 0	1.11 ± 1.11	0 ± 0	0 ± 0	1.67 ± 1.11

Table S3. Percent mortality of medaka embryos exposed to 0.5, 3, or 5 μM 1,2- or 5,6-chrysenequinone (CHQ) from 52-76 hpf. Values represent the mean of three replicates. Error bars represent ± SEM.

	1,2-CHQ			5,6-CHQ		
Maturity	0.5 μM	3 μM	5 μM	0.5 μM	3 μM	5 μM
28 hpf	0 ± 0	0 ± 0	0 ± 0	0 ± 0	0 ± 0	0 ± 0
52 hpf	0 ± 0	0 ± 0	0 ± 0	0 ± 0	0 ± 0	0 ± 0
76 hpf	0 ± 0	1.11 ± 1.11	0 ± 0	0 ± 0	0 ± 0	0 ± 0
100 hpf	1.11 ± 1.11	1.11 ± 1.11	0 ± 0	1.11 ± 1.11	2.22 ± 1.11	0 ± 0
124 hpf	1.11 ± 1.11	1.11 ± 1.11	1.11 ± 1.11	1.11 ± 1.11	2.22 ± 1.11	1.11 ± 1.11
148 hpf	1.11 ± 1.11	1.11 ± 1.11	1.11 ± 1.11	1.11 ± 1.11	2.22 ± 1.11	1.11 ± 1.11
172 hpf	1.11 ± 1.11	1.11 ± 1.11	1.11 ± 1.11	1.11 ± 1.11	2.22 ± 1.11	1.11 ± 1.11

Table S4. Information for chemicals examined in this study.

Chemical	Abbreviation	Structure	CAS No.	LogKow
Chrysene	CHR	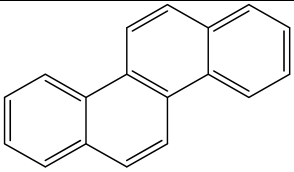	218-01-9	5.7
2-hydroxychrysene	2-OHCHR	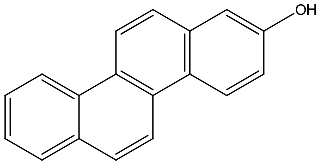	65945-06-4	5.2
6-hydroxychrysene	6-OHCHR	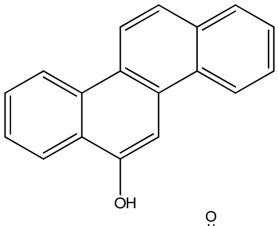	37515-51-8	5.3
1,2-chrysenequinone	1,2-CHQ	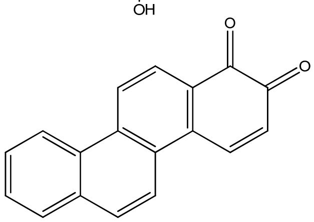	2304-83-8	4.0
1,2-chrysenediol	1,2-CAT	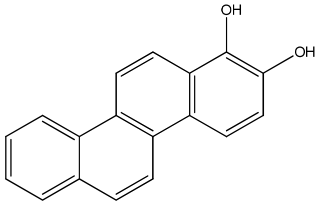	138142-87-7	5.0
5,6-chrysenequinone	5,6-CHQ	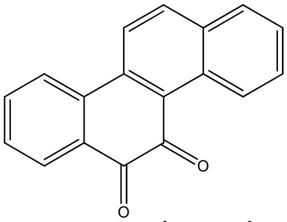	.2051-10-7	3.8
5,6-chrysenediol	5,6-CAT	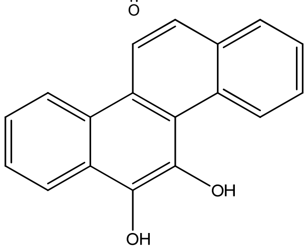	129983-41-1	5.0
2,8-chrysenediol	2,8-DHC	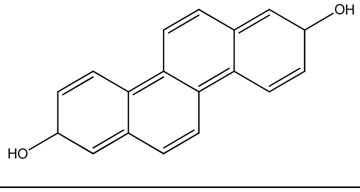	NA	4.6

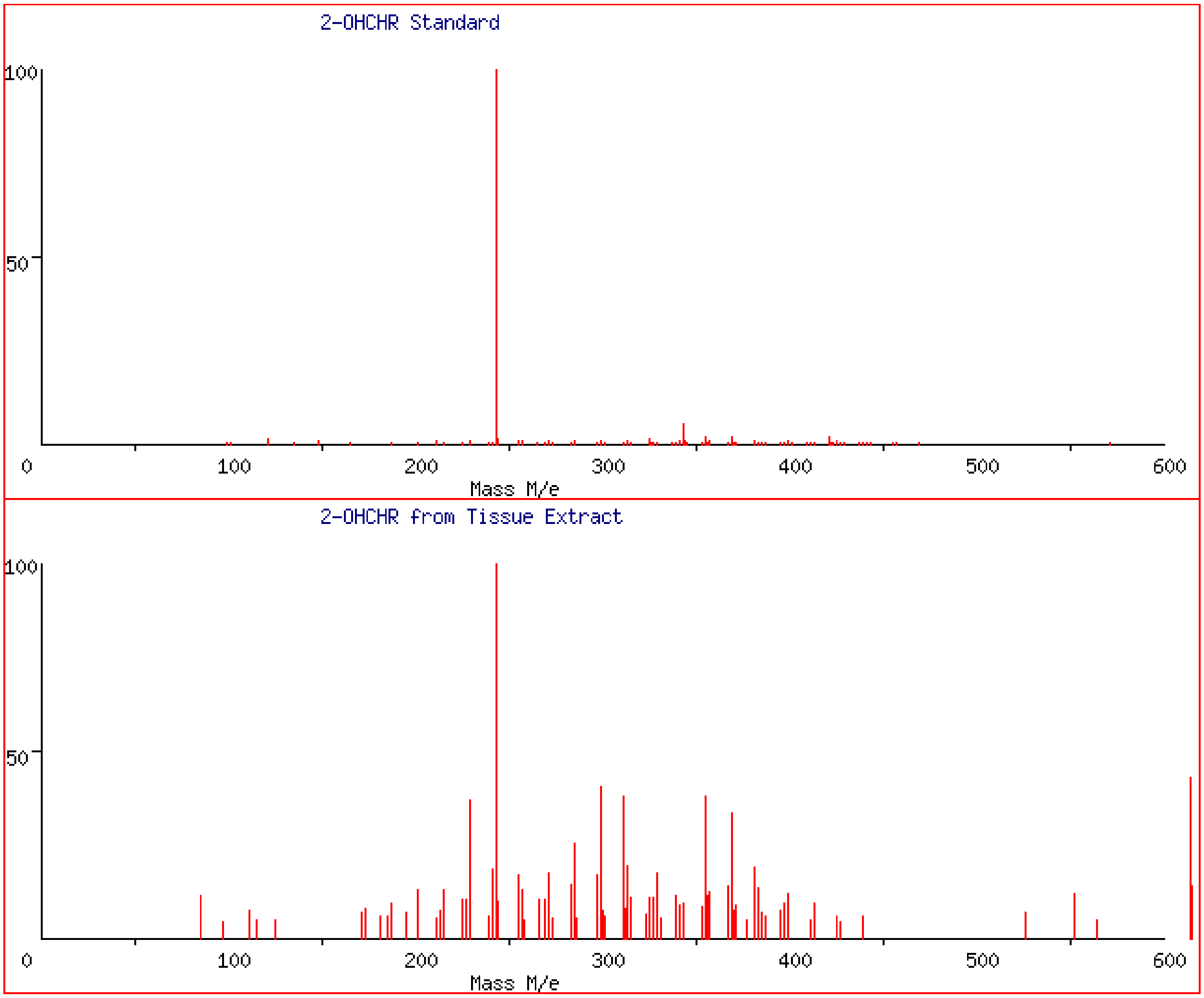

Figure S1. High resolution mass spectra of 2-OHCHR from analytical standards compared to tissue extracts after exposure to 2-OHCHR from 52-76 hpf. Samples were run in negative ion mode.

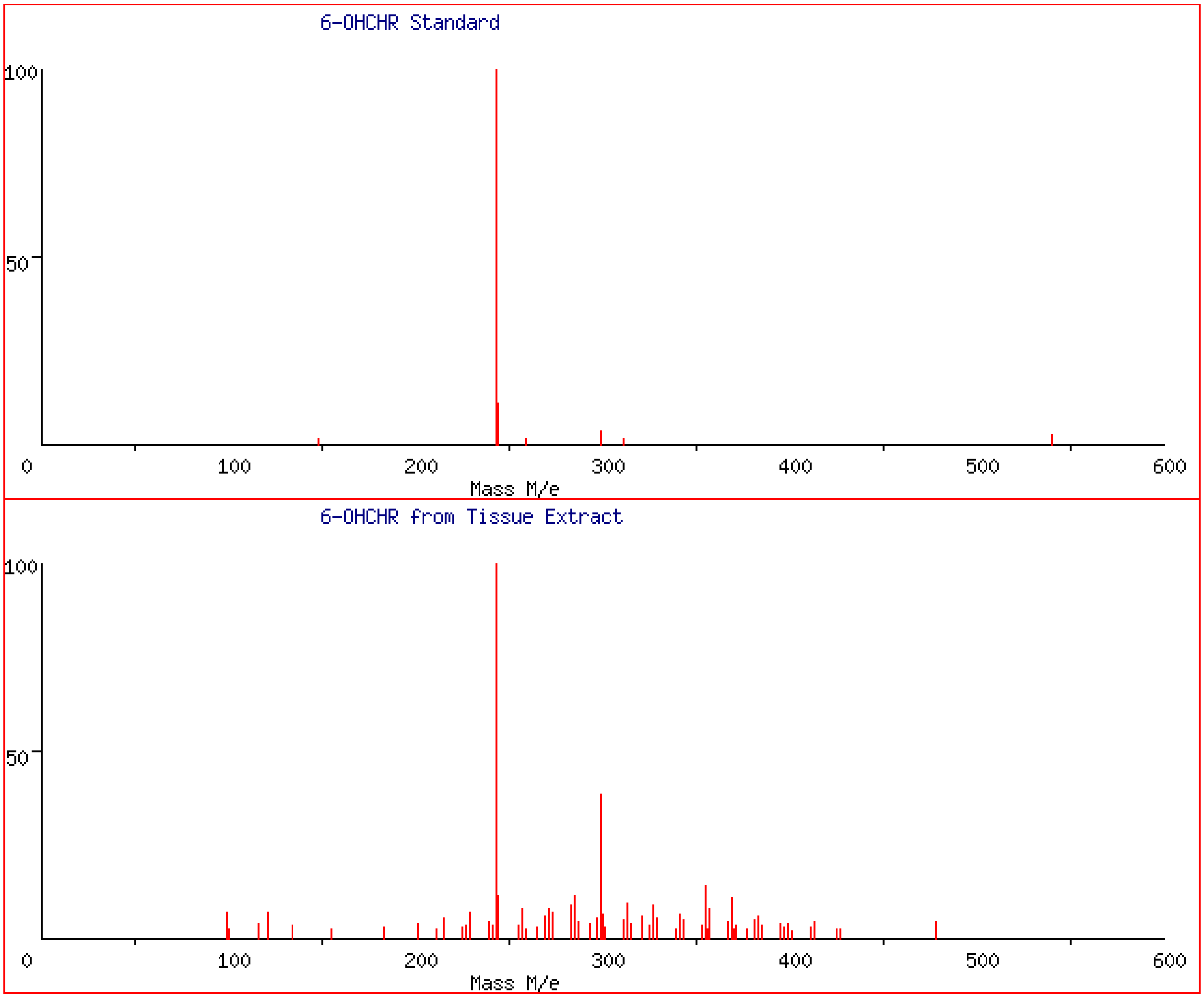

Figure S2. High resolution mass spectra of 6-OHCHR from analytical standards compared to tissue extracts after exposure to 6-OHCHR from 52-76 hpf. Samples were run in negative ion mode.

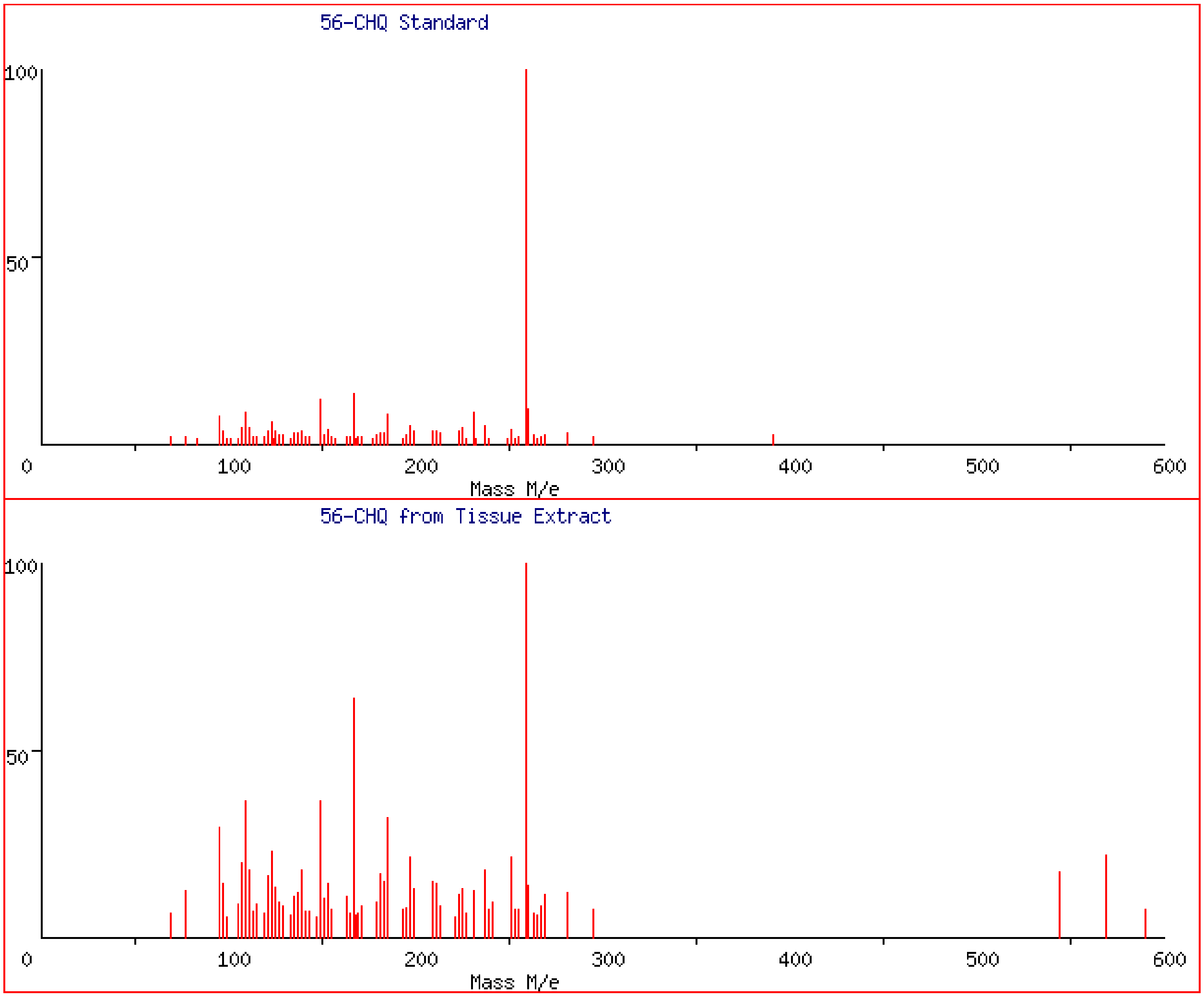

Figure S3. High resolution mass spectra of 5,6-CHQ from analytical standards compared to tissue extracts after exposure to 6-OHCHR from 52-76 hpf. Samples were run in positive ion mode.

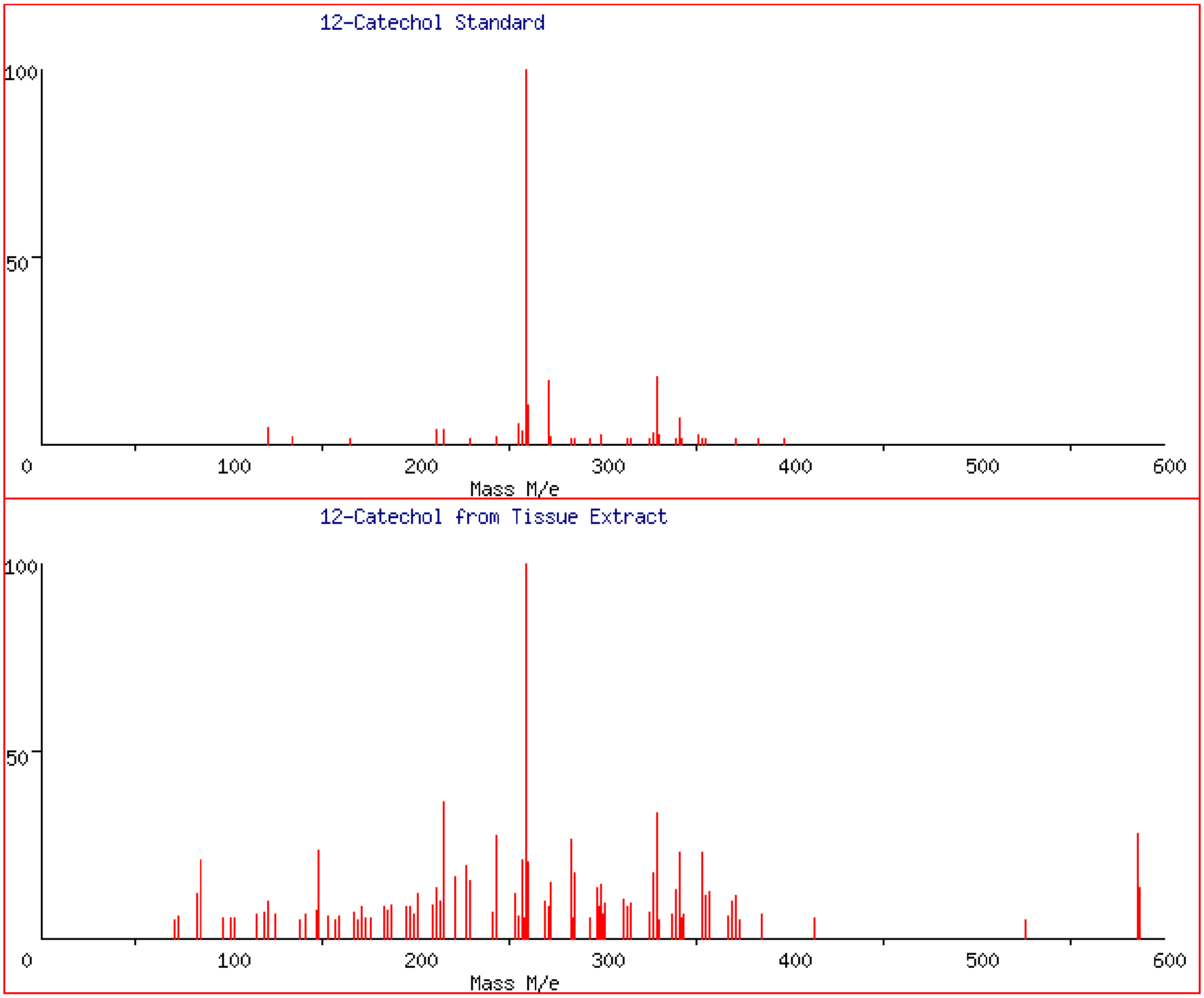

Figure S4. High resolution mass spectra of the 1,2-Catechol of chrysene compared to tissue extracts after exposure to 2-OHCHR from 52-76 hpf. Samples were run in negative ion mode.

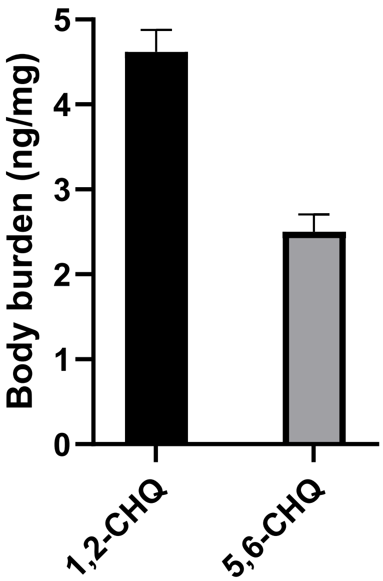

Figure S5. Body burdens of 1,2- or 5,6-CHQ in medaka embryos after exposure to 3 μM from 52-76 hpf.

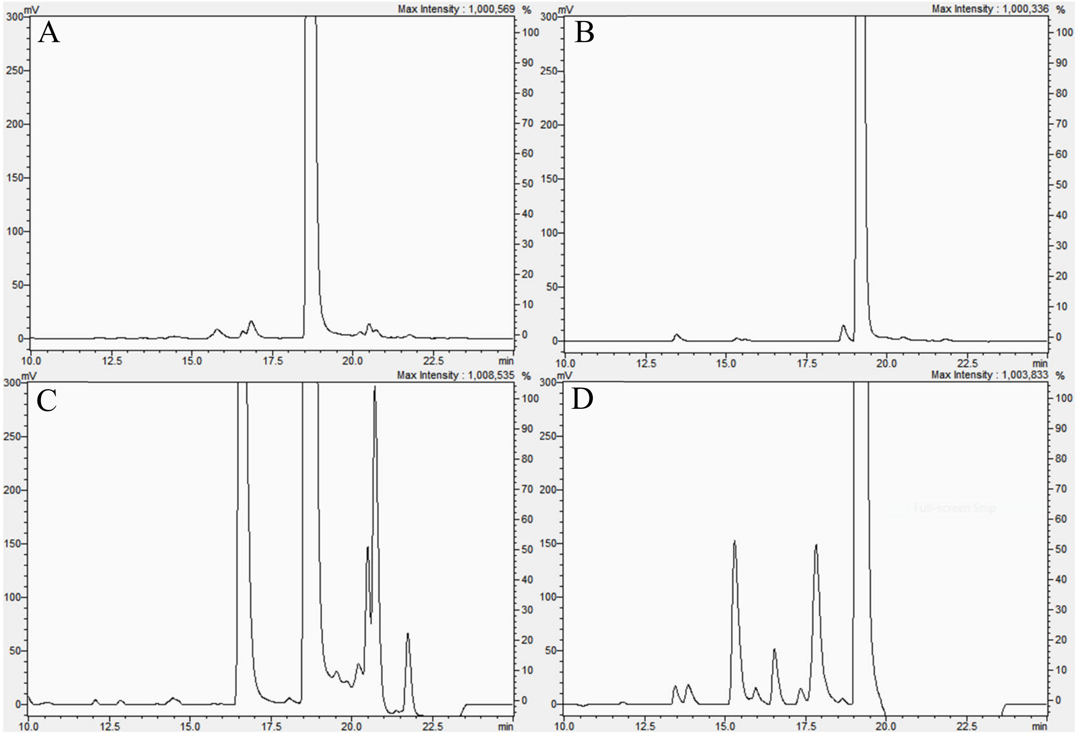

Figure S6. Chromatograms of tissue extracts of embryos exposed from 52-76 hpf 2- or 6-OHCHR. A and B are 2- and 6-OHCHR extracts after 24 h uptake without glucuronidase or sulfatase treatment. C and D are the same treatments but with glucuronidase and sulfatase.

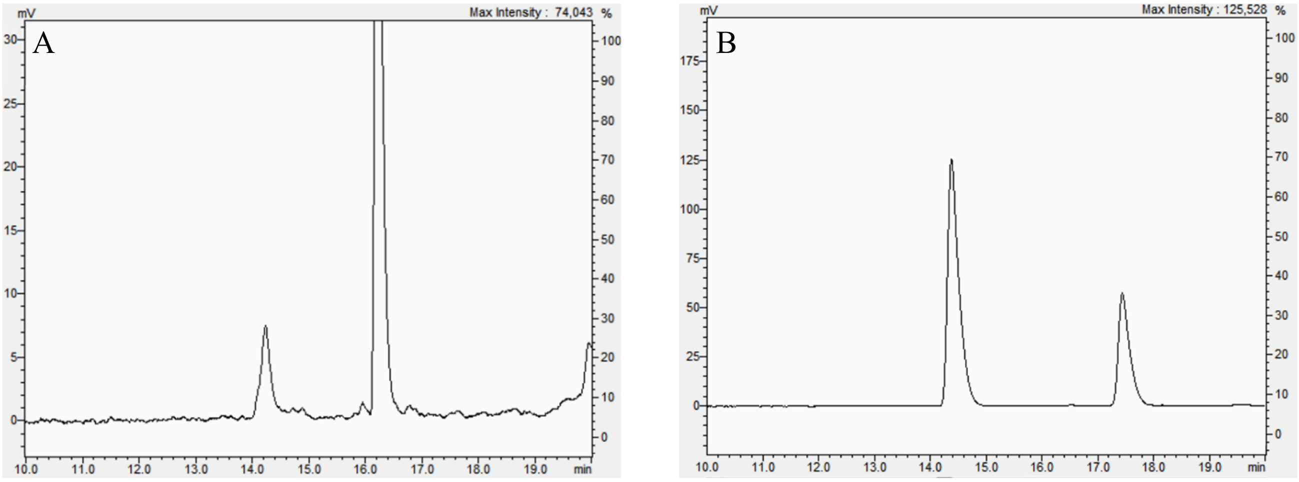

Figure S7. Fluorescence chromatograms of sodium borohydride reductions of 1,2- (A) and 5,6-chrysenequinone (B). The right peak is the catechol while the left is suspected to be a dihydrodiol.

Text S1. Sodium borohydride reduction method

To a glass test tube, 1 mL methanol and 0.5 mL quinone (5 ppm in methanol) were added. Sodium borohydride (50 mg) was then added to the tube and stirred. After 5 minutes, 1 mL water and 1 mL 3N HCl was added. The reduced products were then extracted with 1 mL dichloromethane, twice, then dried with magnesium sulfate. The dichloromethane was then transferred to a glass scintillation vial, dried under a stream of nitrogen gas, then reconstituted with 1 mL 0.1% FA acetonitrile.

Text S2. Mass spectrometric identification

The catechol peaks were detected on a fluorometer and eluted close to the quinone standards. However, quinones fluoresce poorly. In addition, the catechol peaks co-eluted with $NaBH_4$ reduced quinone standards. Thus, we identified these peaks as catechol metabolites. In addition, the masses of the putative catechol metabolites in the extracts were over 99.999% similar to the exact theoretical mass using selected ion monitoring high resolution mass spectrometry.

Part 2: Synthesis of 1,2-dihydroxychrysene (1,2-catechol)

The photochemical reactions were performed with in Photochemical Reactors Ltd. 400 W medium pressure Mercury-lamp in a 2 L quartz immersion well reactor fitted with a no. 3408 glass filter sleeve. All reactions were carried out under N_2 and in oven-dried glassware.

NMR: Spectra were recorded on a 400 MHz Bruker AVANCE III spectrometer; chemical shifts (δ) are given in ppm relative to the TMS signal (0.00 ppm) for ^{1}H NMR and the solvent signal for ^{13}C NMR ($CDCl_3$ at 77.0 ppm and aceton-d_6 at 29.8 ppm). All NMR spectra were processed using Topspin NMR software. Melting points were measured on Büchi MP-3 melting point apparatus (uncorrected). HRMS were measured on an orbitrap exploris 120 with a APCI probe by Thermo Fischer Scintific by senior engineer Jostein A. Johansen at the University of Tromsø.

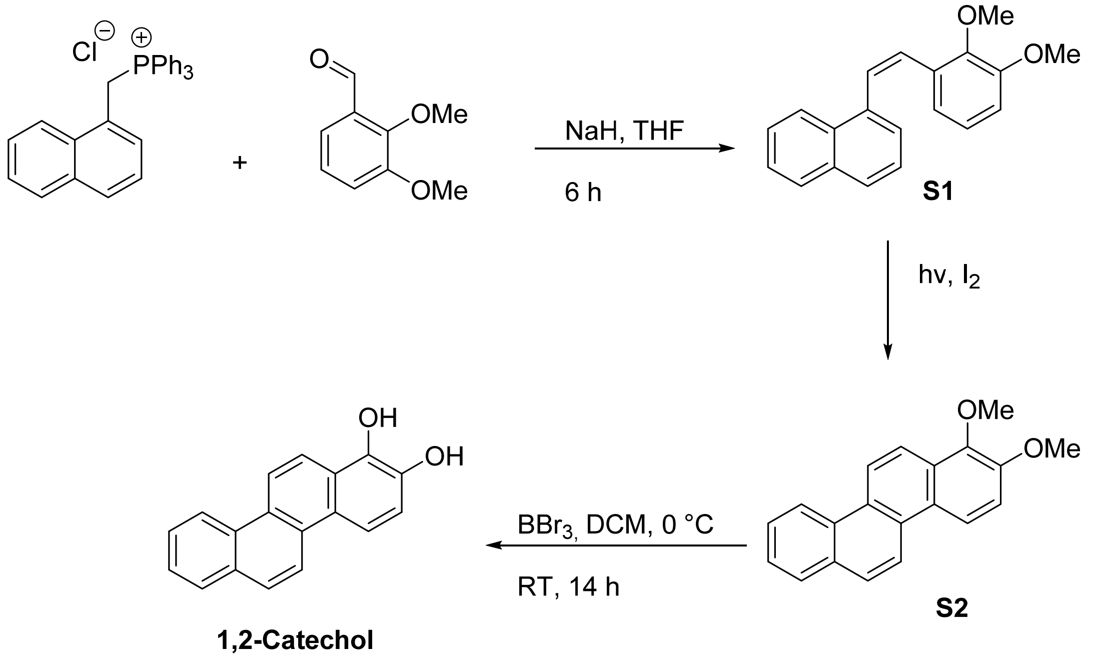

Scheme 1: Photochemical synthesis of 1, 2-dihydroxychrysene (1,2-catechol).

(2,3-dimethoxystyryl) naphthalene (**S1**): 2,3-dimethoxybenzaldehyde (4.00 g, 24.1 mmol) and (naphthalen-1-ylmethyl)triphenyl phosphonium chloride[S1] (1.2 equiv., 12.7 g, 28.9 mmol) were dissolved in THF (210 mL) and added NaH (1.5 Equiv., 0.87 g, 36.1 mmol) at 0 °C. The reaction mixture was refluxed for 6 h. before cooled down and carefully quenched with cold water (100 mL). The water phase was further extracted with diethyl ether (100 mL) and the combined organic layers were dried over Na_2SO_4 and concentrated under reduced pressure. The remaining oil was purified by flash chromatography (heptane: ethyl acetate 9:1) to give *cis/trans*-$\mathbf{S_1}$ (6.48 g, 93%) as a thick yellow oil.

1,2- dimethoxychrysene (**S2**): Stilbene **S1** (3.50 g, 12.1 mmol), I_2 (1.2 equiv., 3.67 g, 14.5 mmol) and 1,2-epoxybutane (30 equiv., 30 mL) was dissolved in degassed toluene (1.2 L) in the photochemical reactor. This reaction mixture was irradiated under nitrogen atmosphere for 14 h.

The reaction mixture was reduced to 300 mL under reduced pressure, washed with 10% aqueous $Na_2S_2O_3$ (150 mL) followed by brine (80 mL). The organic dried over anhydrous Na_2SO_4 and concentrated under reduced pressure. The remains were purified by flash chromatography (heptane: ethyl acetate 4:1) to afford 2.16 g (62%) of **S1** as white crystals. Mp. 206-207 °C (heptane).

^{1}H NMR (400 MHz, $CDCl_3$) δ: 4.06 (s, 3H), 4.07 (s, 3H), 7.44 (d, *J* = 9.2 Hz, 1H), 7.61 (td, *J* = 7.4, 1.1 Hz, 1H), 7.69 (td, *J* = 7.6, 1.4 Hz, 1H), 7.97 (d, *J* = 98.8 Hz, 2H), 8.33 (d, *J* = 9.4 Hz, 1H), 8.50 (d, *J* = 9.1, 1H), 8.62 (d, *J* = 9.1 Hz, 1H); 8.71 (d, *J* = 9.3 Hz, 1H); 8.76 (d, *J* = 9.2 Hz, 1H) ppm; ^{13}C NMR (100 MHz, $CDCl_3$) δ: 56.6, 61.4, 114.3, 119.3, 120.7, 121.1, 121.7, 123.0, 126.1, 126.13, 126.7, 126.9, 127.4, 127.8, 128.3, 128.5, 130.6,131.9, 143.6,149.3 ppm. HRMS (APCI) *m/z*: calcd. for $C_{18}H_{13}O_2$ $[M + H]^+$: 289.1223; found, 289.1223.

Chrysene-1,2-diol (**1,2-Catechol**)[S2]: To a stirred solution of **S2** (880 mg, 3.05 mmol) in DCM (90 mL), a solution of boron tribromide (3 equiv., 0.87 mL, 9.15 mmol) was added slowly through a septum with a syringe at -25 °C under nitrogen atmosphere. The cold bath was removed, and the mixture was stirred for 30 min. Ice-cold water (100 mL) was added through a syringe and the organic layer was separated, washed with water (3 x 100 mL), and dried over Na_2SO_4. The solvent was removed under reduced pressure and recrystallization the solid residue with acetonitrile: heptane (1:2) to afford 510 mg (64%) of **1,2-Catechol**. M.p 289-292 °C (dec.) (Lit [S2]: 295-297 °C (dec.)).

^{1}H NMR (400 MHz, acetone-d_6) δ: 7.26 (d, *J* = 9.0 Hz, 1H), 7.49 (td, *J* = 7.4, 1.1 Hz, 1H), 7.59 (td, *J* = 7.6, 1.4 Hz, 1H), 7.90 (d, *J* = 9.2 Hz, 1H), 8.18 (d, *J* = 8.8 Hz, 1H), 8.27 (dd, *J* = 9.3, 0.5 Hz, 1H), 8.60 (d, *J* = 9.2 Hz, 1H); 8.63 (d, *J* = 9.3 Hz, 1H); 8.75 (d, *J* = 8.2 Hz, 1H) ppm;^{13}C NMR (100 MHz,) δ: 114.8, 117.3, 117.4, 120.1, 120.7, 121.4, 123.0, 125.4, 125.9, 126.5, 126.7, 128.4, 130.5, 131.8, 139.2,141.0 ppm; HRMS (ESI) *m/z*: calcd. for $C_{18}H_{12}O_2$, 259.0759 $[M - H]^+$; found, 259.0762.

^{1}H NMR data are similar to those reported in dmso-d_6 by Klein *et al*. [S2]

1,2- dimethoxychrysene (**S2**)

^{1}H-NMR (400 MHz, $CDCl_3$)

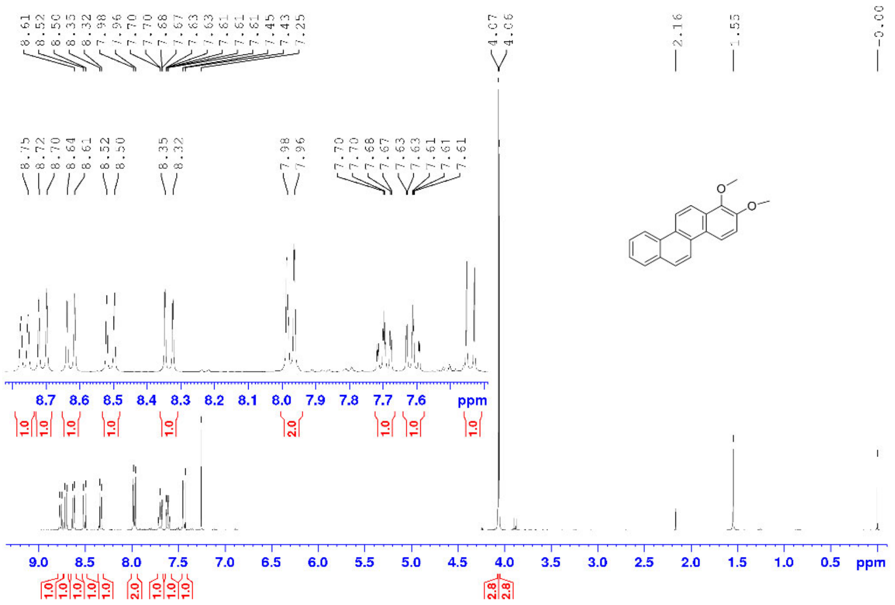

^{13}C{1H}-NMR (100 MHz, CDCl3)

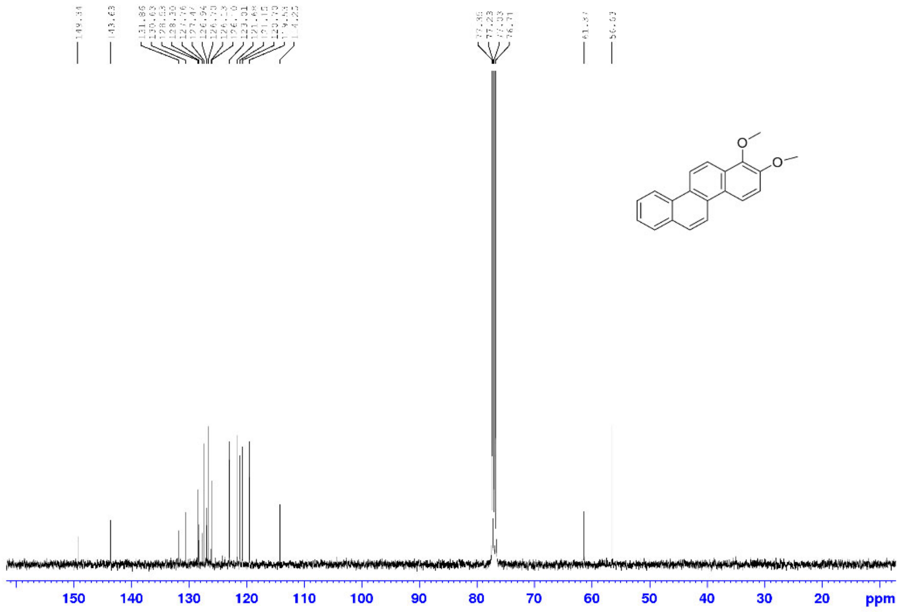

Chrysene-1,2-diol (**1,2-Catechol**)

^{1}H-NMR (400 MHz, acetone-d_6)

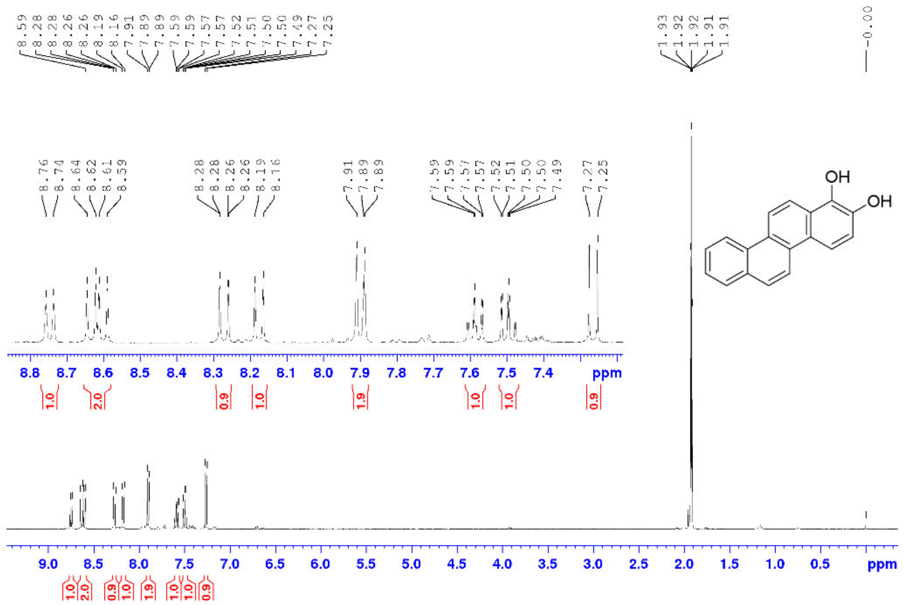

^{13}C- NMR (100 MHz, acetone-d_6)

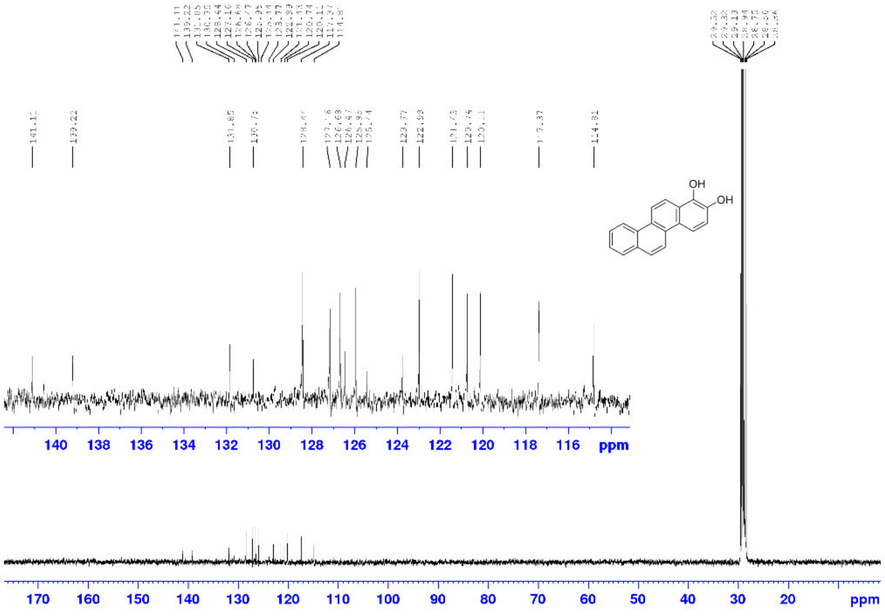

References

[S1] Jørgensen, K. B.; Joensen, M., Photochemical Synthesis of Chrysenols. *Polycyclic Aromatic Compounds* **28** (4-5), 362-372 (2008).

[S2] Klein, J.; Seidel, A.; Frank, H.; Oesch, F.; Platt, K. L., Regiospecific oxidation of polycyclic aromatic dihydrodiols by rat liver dihydrodiol dehydrogenase. *Chemico-biological interactions* **79** (3), 287-303 (1991).

Part 3: Synthesis of 2,8-dihydroxychrysene

Synthesis of 2,8-Dihydroxychrysene (6)

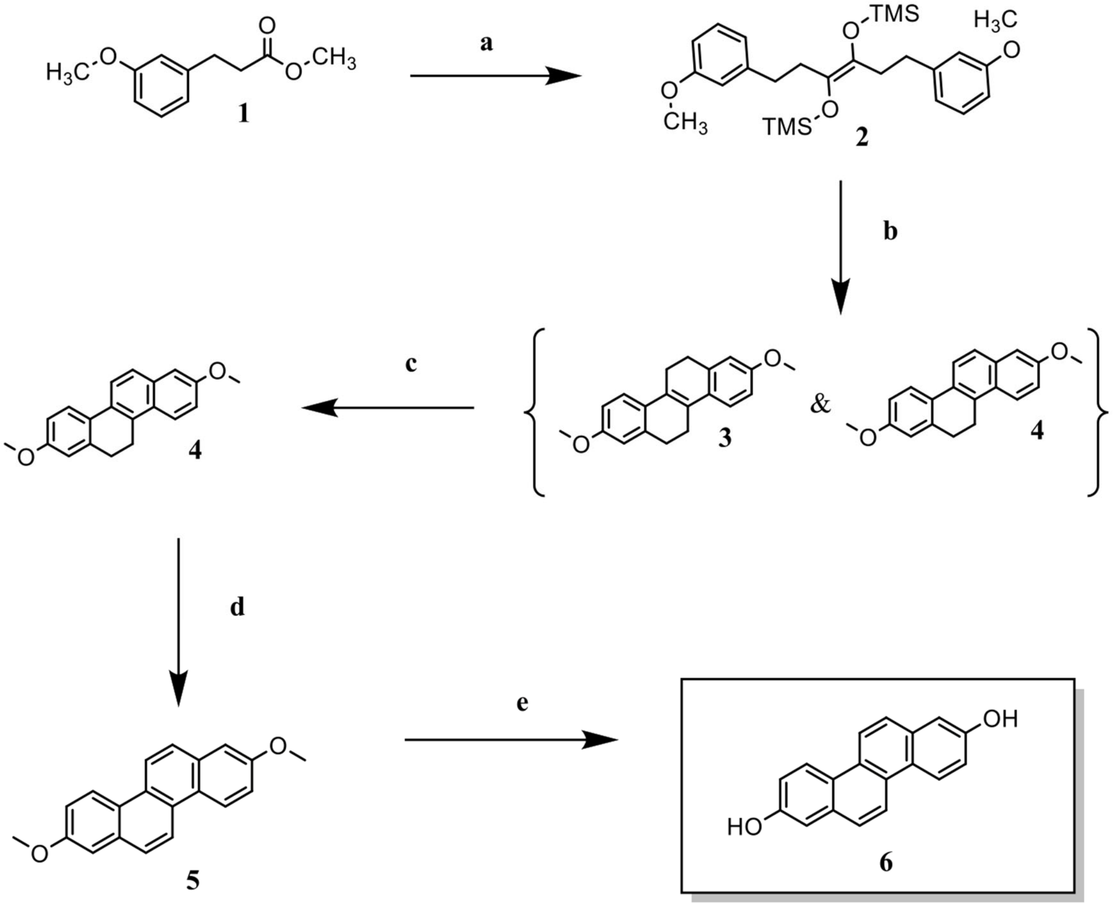

Scheme 1: Synthesis of 2,8-dihydroxychrysene (**6**). Reagents and conditions: (a) Sodium, TMSCl, toluene, 120°C, 18 h; (b) PPA, rt, 3 h; (c) Pd/C, mesitylene, 160°C, 6 h; (d) Sulfur, neat, 180-200°C, 2 h; (e) BBr_3, -78°C to rt, 22 h.

2,8-dihydroxychrysene (**6**) was synthesized according to a combination of published procedures, with modification[1,2]. Briefly, commercially available ester **1** was converted to the silyl ether **2** and then subjected to an acyloin condensation reaction affording a mixture of 2,8-dimethoxy-tetrahydrochrysenes (**3** and **4**). This mixture was oxidized to a signal isomer with Pd/C to afford 2,8-dimethoxy-dihydrochrysene **4**, further oxidized to the fully aromatic compound **5** with elemental sulfur. The methyl group deprotection was accomplished with boron tribromide to yield the target compound 2,8-dihydroxychrysene (**6**).

General Experimental Procedures: Chrysene was purchased from MedChemExpress LLC (Monmouth Junction, NJ). Polyphosphoric acid, ca 84% was purchased from Alfa Aesar (Ward Hill, MA). 1,3,5-Trimethylbenzene 97.0+% was purchased from TCI America. Other reagents and solvents used, including sodium sticks coated in film of protective hydrocarbon oil 99%, toluene anhydrous 98.8%, and sulfur pieces 99.99%, were purchased from Fisher Scientific (Hanover Park, IL). NMR analysis was performed in deuterated solvents on a Bruker 400 MHz spectrometer. Chemical shifts are reported relative to the tetramethylsilane internal standard or solvent peaks. Low resolution mass spectrometry was performed on a Waters ZQ single quad instrument in ESI+ or ESI- mode.

<u>4,5-bis(3-methoxyphenethyl)-2,2,7,7-tetramethyl-3,6-dioxa-2,7-disilaoct-4-ene (2):</u>

Sodium metal (1.25 g, 54.4 mmol) was quickly added to a vacuum-dried and argon-purged round bottom flask containing anhydrous toluene (85 mL). The mixture was heated to reflux and stirred vigorously for 30 minutes. Immediately upon reaching 120°C, sodium dispersed into fine granules. Methyl-3-(3-methoxyphenyl) propanoate (**1**) (3.0 g, 15.5 mmol) was mixed with trimethylsilyl chloride (6 mL, 46 mmol) in anhydrous toluene (5 mL); this solution was added dropwise via syringe to the sodium mixture. The yellow solution was subsequently heated for 18 hours at reflux. After cooling, precipitates were removed by filtration. The clear filtrate was washed successively with 1 N HCl, water, and brine followed by organic extraction into diethyl ether (50 mL). The organic collection was dried over sodium sulfate, filtered, and concentrated under reduced pressure to afford the yellow crude silyl ether 2 (3.6 g, 78%). The silyl ether was used without further purification. ESI-MS calculated for $C_{26}H_{40}O_4Si_2$: MH^+/z 495.2 as Na^+ adduct; Found: MH^+/z 495.0. ^{1}H NMR (400 MHz, Chloroform-*d*) δ 7.27 – 7.17 (m, 2H), 6.86 – 6.70 (m, 6H), 3.85 – 3.76 (m, 6H), 2.99 – 2.65 (m, 4H), 2.64 – 2.24 (m, 4H), 0.28 – 0.12 (m, 18H).

<u>Acyloin condensation of 2: Synthesis of tetrahydrochrysene analogs 3 and 4:</u>

Polyphosphoric acid (PPA, 38 g) was transferred into an Erlenmeyer flask equipped with a large stir bar and silyl ether **2** (2.6 g) was added, immediately reddening at the interface. Due to the high viscosity of PPA, the mixture was stirred with a strong magnet for 3 hours at room temperature. Over the course of the reaction, the mixture shifted in color from bright to dark red, then red-orange upon completion. After 3 hours of stirring, the mixture was allowed to rest without stirring overnight at room temperature. Water (75 mL) was carefully added to the reaction mixture generating a pink suspension. To the suspension, ethyl acetate was added until all solids were dissolved (150 mL). The organic fractions were washed successively with saturated sodium

bicarbonate, brine and extracted. The organic phases were dried over sodium sulfate and concentrated under reduced pressure to give a sticky tan paste. Crystallization from ethyl acetate afforded a pale yellow solid mixture of **3** and **4** (1.541 g, 95%); ^{1}H NMR (400 MHz, $CDCl_3$) δ 8.03 (d, *J* = 9.1 Hz, 1H), 7.85 (d, *J* = 8.7 Hz, 1H), 7.71 (dd, *J* = 11.3, 8.5 Hz, 2H), 7.27 – 7.19 (m, 3H), 7.19 – 7.15 (m, 2H), 6.92 – 6.84 (m, 2H), 6.76 (d, *J* = 7.8 Hz, 4H), 3.95 (s, 3H), 3.87 (s, 3H), 3.83 (s, 6H), 3.25 (t, *J* = 7.4 Hz, 2H), 2.97 (t, *J* = 7.3 Hz, 2H), 2.89 (t, *J* = 7.6 Hz, 4H), 2.65 (t, *J* = 7.3 Hz, 4H).

2,8-Dimethoxy-5,6-dihydrochrysene (4):

The mixture of **3** and **4** (500 mg) was suspended in mesitylene (35 mL) and heated to 100°C, with stirring. Then, a 10% w/w palladium on carbon (251.2 mg) was added. The reaction flask was equipped with a reflux condenser and moisture trap, then the mixture was heated to 160°C. After 6 hours, the mixture was filtered via sintered glass funnel immediately upon removing it from heat to prevent precipitation prior to filtration. Gray precipitates quickly formed in the filtrate. The filtrate was stored at -20°C for 3 days to allow for complete precipitation. The mixture was decanted to remove mesitylene, and solids were rinsed with hexanes. The collected solids were redissolved in warm THF, filtered, and solvent was removed under reduced pressure to afford approximately 100 mg of **4** (20%). m.p.207-209°C; ^{1}H NMR (400 MHz, $CDCl_3$) δ 8.01 (d, *J* = 9.3 Hz, 1H), 7.83 (d, *J* = 9.0 Hz, 1H), 7.69 (t, *J* = 10.1 Hz, 2H), 7.31 (s, 1H), 7.27 – 7.23 (m, 1H), 7.20 – 7.12 (m, 2H), 6.90 – 6.82 (m, 2H), 3.99 (q, *J* = 2.1 Hz, 1H), 3.93 (q, *J* = 2.1 Hz, 3H), 3.86 (q, *J* = 2.0 Hz, 3H), 3.24 (s, 2H), 2.96 (d, *J* = 7.6 Hz, 2H).

2,8-Dimethoxychrysene (5):

To a flask containing **4** (100 mg, 0.3 mmol) was added an excess amount of crushed sulfur (approximately 180 mg). The solid mixture was heated to 180-200°C for 2 hours without stirring, which caused the mixture to melt and eventually blacken. The resulting char was reconstituted in toluene with stirring at 100°C. The solids were allowed to settle, the mixture was decanted, and the solids were rinsed with hexanes to afford **5** as a grey solid (~80 mg, 81%). ^{1}H NMR (400 MHz, $CDCl_3$) δ 8.63 (dd, *J* = 13.7, 9.1 Hz, 4H), 7.90 (d, *J* = 9.0 Hz, 2H), 7.34 (d, *J* = 8.2 Hz, 4H), 3.99 (s, 6H).

2,8-Dihydroxychrysene (6):

Compound **5** (65 mg) was suspended in anhydrous dichloromethane (5 mL) and cooled to -78°C while stirring under argon atmosphere. Boron tribromide (approximately 50 µL, neat) was added dropwise via syringe. The reaction was allowed to slowly warm to room temperature and was left to stir overnight (22 h). The reaction mixture was cooled on ice while adding saturated sodium bicarbonate solution (10 mL) dropwise with continued stirring. The mixture was transferred to a 20 mL scintillation vial and remaining solids were transferred as a suspension. The solids were allowed to settle overnight to allow for effective decanting of the aqueous layer. The remaining solids were washed a second time with dilute sodium bicarbonate (10 mL) and the aqueous fraction was again decanted after sufficient settling time. Residual solvent was removed overnight under

high vacuum to afford the off-white solid **6** (58 mg, 99%). ESI-MS (negative ion mode) calculated for $C_{18}H_{12}O_2$: M-H/z 259.1; Found: M-H/z 259.2. m.p.314°C-316°C; ^{1}H NMR (400 MHz, DMSO-d_6) δ 9.89 (s, 2H), 8.65 (dd, J = 21.1, 9.1 Hz, 4H), 7.85 (d, J = 9.1 Hz, 2H), 7.41 – 7.05 (m, 4H); ^{13}C NMR (400 MHz, DMSO-d_6) δ 156.26, 133.36, 126.81, 126.57, 125.08, 124.22, 121.89, 118.63, 111.00, 40.41, 40.20, 39.99, 39.78, 39.58.

References

1. Hwang, K. J., O'Neil, J. P., & Katzenellenbogen, J. A. (1992). 5, 6, 11, 12-Tetrahydrochrysenes: synthesis of rigid stilbene systems designed to be fluorescent ligands for the estrogen receptor. The Journal of Organic Chemistry, 57(4), 1262-1271.

2. Johnson, W. S., Erickson, C. A., & Ackerman, J. (1952). 2, 8-Dihydroxy-5, 6, 11, 12-tetrahydrochrysene. Journal of the American Chemical Society, 74(9), 2251-2253.

Spectral Data

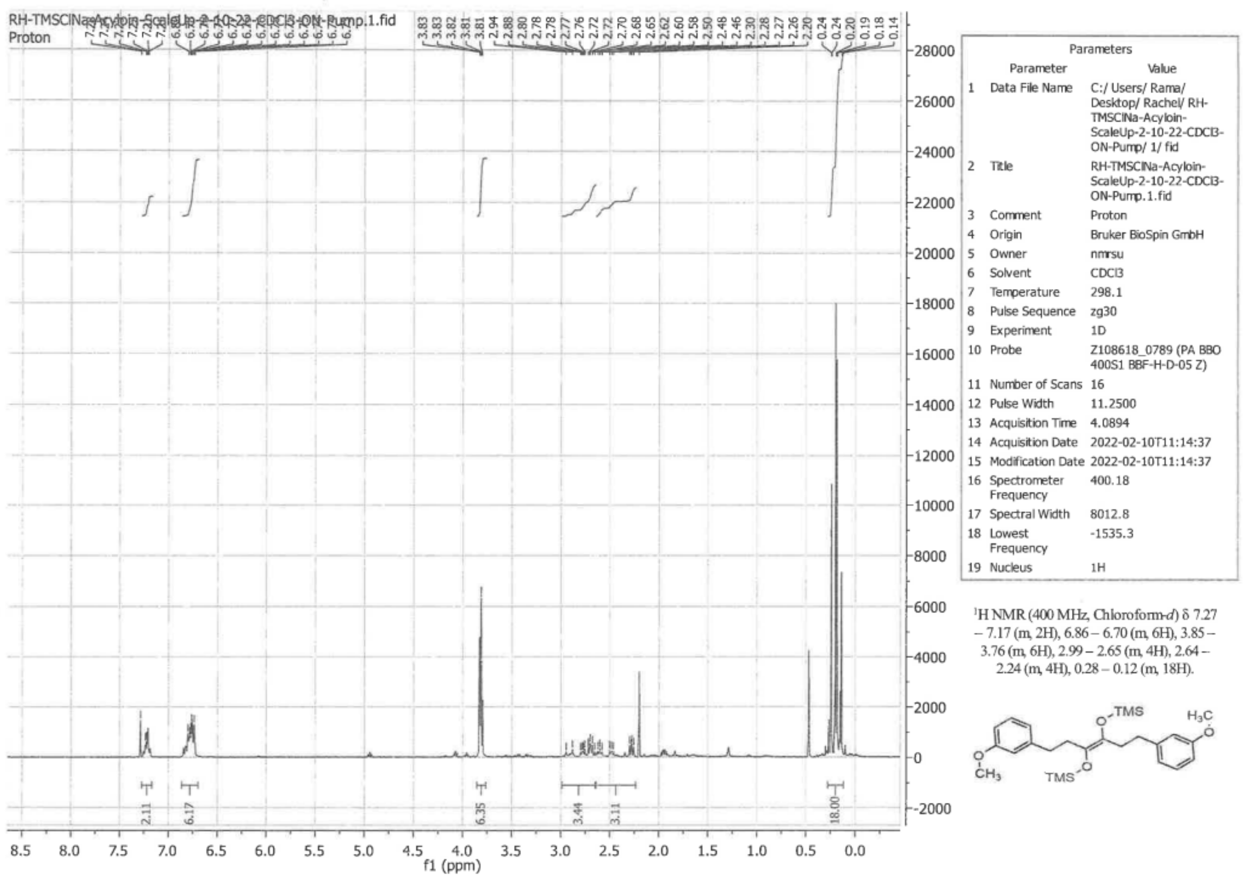

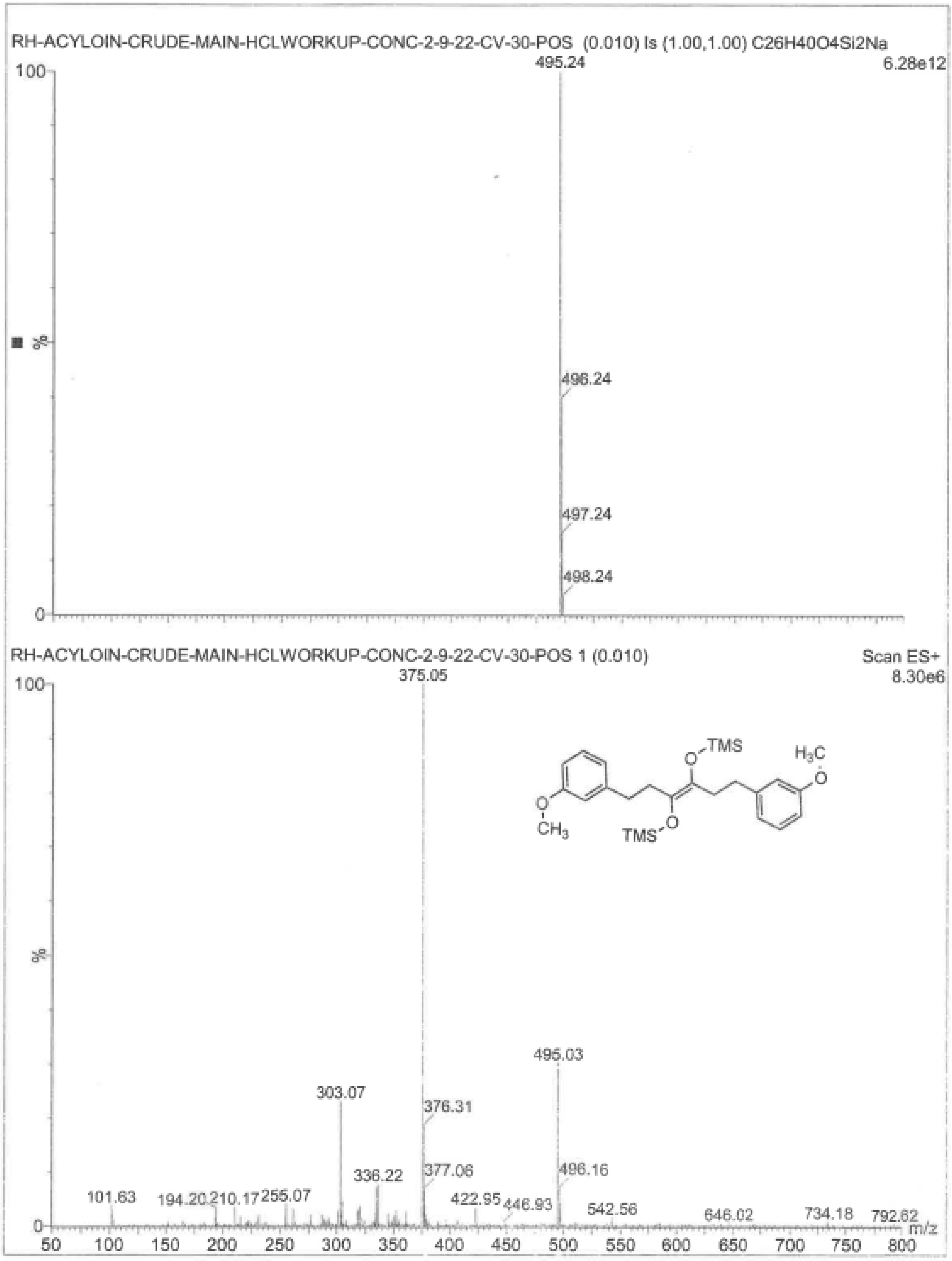
RH-ACYLOIN-CRUDE-MAIN-HCLWORKUP-CONC-2-9-22-CV-30-POS (0.010) Is (1.00,1.00) C26H40O4Si2Na
6.28e12
495.24
496.24
497.24
498.24
RH-ACYLOIN-CRUDE-MAIN-HCLWORKUP-CONC-2-9-22-CV-30-POS 1 (0.010)
Scan ES+
8.30e6
375.05
495.03
303.07
376.31
336.22
377.06
496.16
101.63
194.20
210.17
255.07
422.95
446.93
542.56
646.02
734.18
792.62
m/z
%
TMS
H3C
CH3

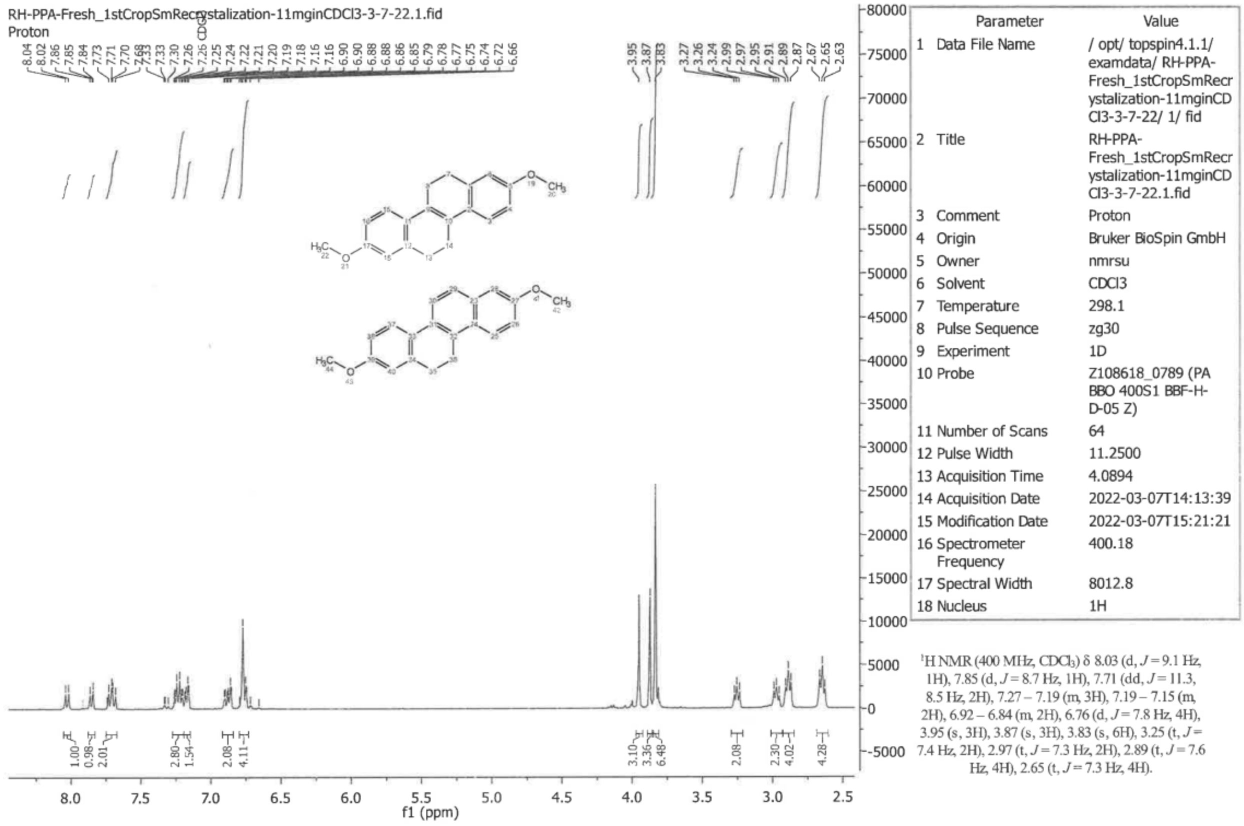

	Parameter	Value
1	Data File Name	/ opt/ topspin4.1.1/ examdata/ RH-PPA-Fresh_1stCropSmRecrystalization-11mginCDCl3-3-7-22/ 1/ fid
2	Title	RH-PPA-Fresh_1stCropSmRecrystalization-11mginCDCl3-3-7-22.1.fid
3	Comment	Proton
4	Origin	Bruker BioSpin GmbH
5	Owner	nmrsu
6	Solvent	CDCl3
7	Temperature	298.1
8	Pulse Sequence	zg30
9	Experiment	1D
10	Probe	Z108618_0789 (PA BBO 400S1 BBF-H-D-05 Z)
11	Number of Scans	64
12	Pulse Width	11.2500
13	Acquisition Time	4.0894
14	Acquisition Date	2022-03-07T14:13:39
15	Modification Date	2022-03-07T15:21:21
16	Spectrometer Frequency	400.18
17	Spectral Width	8012.8
18	Nucleus	1H

^{1}H NMR (400 MHz, $CDCl_3$) δ 8.03 (d, J = 9.1 Hz, 1H), 7.85 (d, J = 8.7 Hz, 1H), 7.71 (dd, J = 11.3, 8.5 Hz, 2H), 7.27 – 7.19 (m, 3H), 7.19 – 7.15 (m, 2H), 6.92 – 6.84 (m, 2H), 6.76 (d, J = 7.8 Hz, 4H), 3.95 (s, 3H), 3.87 (s, 3H), 3.83 (s, 6H), 3.25 (t, J = 7.4 Hz, 2H), 2.97 (t, J = 7.3 Hz, 2H), 2.89 (t, J = 7.6 Hz, 4H), 2.65 (t, J = 7.3 Hz, 4H).

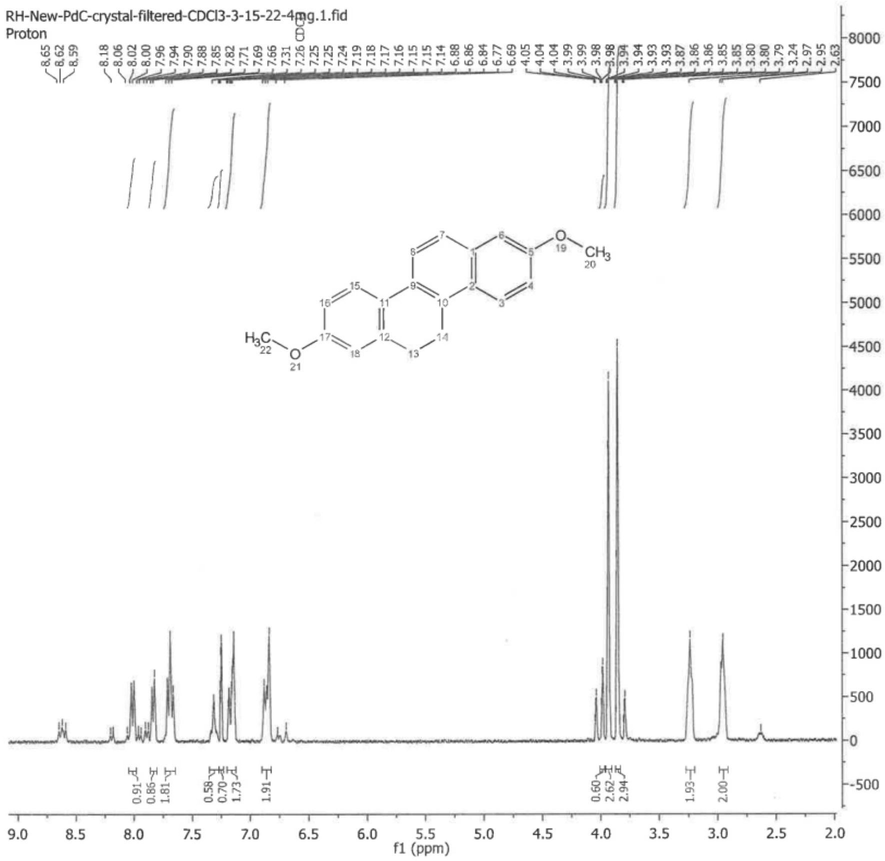

	Parameter	Value
1	Data File Name	/ opt/ topspin4.1.1/ examdata/ RH-New-PdC-crystal-filtered-CDCl3-3-15-22-4mg/ 1/ fid
2	Title	RH-New-PdC-crystal-filtered-CDCl3-3-15-22-4mg.1.fid
3	Comment	Proton
4	Origin	Bruker BioSpin GmbH
5	Owner	nmrsu
6	Solvent	CDCl3
7	Temperature	298.1
8	Pulse Sequence	zg30
9	Experiment	1D
10	Probe	Z108618_0789 (PA BBO 400S1 BBF-H-D-05 Z)
11	Number of Scans	64
12	Pulse Width	11.2500
13	Acquisition Time	4.0894
14	Acquisition Date	2022-03-15T12:13:17
15	Modification Date	2022-03-21T09:21:18
16	Spectrometer Frequency	400.18
17	Spectral Width	8012.8
18	Nucleus	1H

^{1}H NMR (400 MHz, $CDCl_3$) δ 8.01 (d, J = 9.3 Hz, 1H), 7.83 (d, J = 9.0 Hz, 1H), 7.69 (t, J = 10.1 Hz, 2H), 7.31 (s, 1H), 7.27 – 7.23 (m, 1H), 7.20 – 7.12 (m, 2H), 6.90 – 6.82 (m, 2H), 3.99 (q, J = 2.1 Hz, 1H), 3.93 (q, J = 2.1 Hz, 3H), 3.86 (q, J = 2.0 Hz, 3H), 3.24 (s, 2H), 2.96 (d, J = 7.6 Hz, 2H).

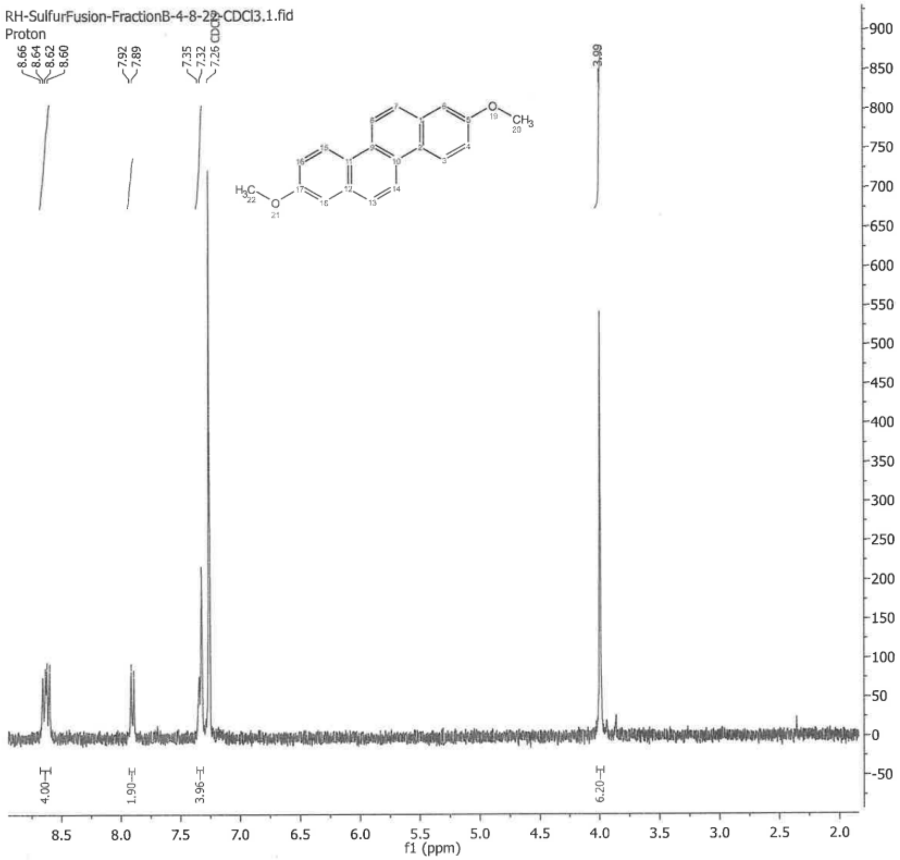

	Parameter	Value
1	Data File Name	/ opt/ topspin4.1.1/ examdata/ RH-SulfurFusion-FractionB-4-8-22-CDCl3/ 1/ fid
2	Title	RH-SulfurFusion-FractionB-4-8-22-CDCl3.1.fid
3	Comment	Proton
4	Origin	Bruker BioSpin GmbH
5	Owner	nmrsu
6	Solvent	CDCl3
7	Temperature	298.1
8	Pulse Sequence	zg30
9	Experiment	1D
10	Probe	Z108618_0813 (PA BBO 400S1 BBF-H-D-05 Z)
11	Number of Scans	32
12	Pulse Width	14.0000
13	Acquisition Time	2.0447
14	Acquisition Date	2022-04-08T14:10:00
15	Modification Date	2022-04-08T14:22:15
16	Spectrometer Frequency	400.13
17	Spectral Width	8012.8
18	Nucleus	1H

^{1}H NMR (400 MHz, $CDCl_3$) δ 8.63 (dd, J = 13.7, 9.1 Hz, 4H), 7.90 (d, J = 9.0 Hz, 2H), 7.34 (d, J = 8.2 Hz, 4H), 3.99 (s, 6H).

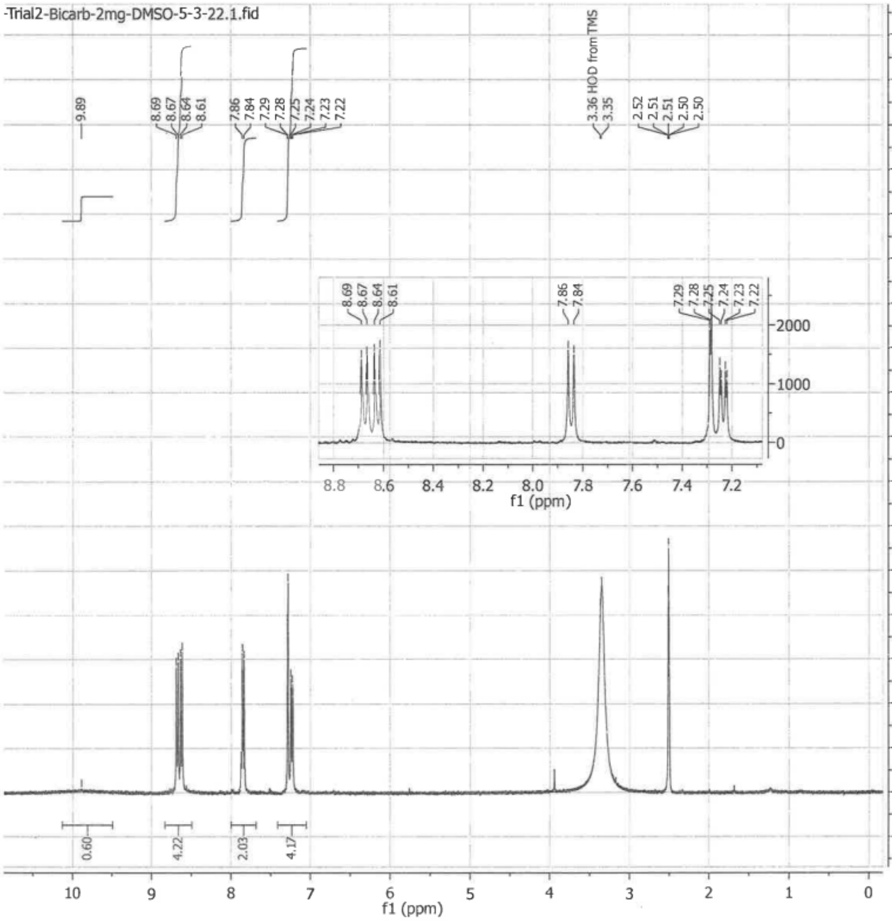

Parameter	Value
1 Data File Name	C:/ Users/ Rama/ Desktop/ Rachel/ RH-BBr3-Trial2-Bicarb-2mg-DMSO-5-3-22/ 1/ fid
2 Title	RH-BBr3-Trial2-Bicarb-2mg-DMSO-5-3-22.1.fid
3 Comment	Proton
4 Origin	Bruker BioSpin GmbH
5 Owner	nmrsu
6 Solvent	DMSO
7 Temperature	298.1
8 Pulse Sequence	zg30
9 Experiment	1D
10 Probe	Z108618_0813 (PA BBO 400S1 BBF-H-D-05 Z)
11 Number of Scans	70
12 Pulse Width	10.0000
13 Acquisition Time	4.0894
14 Acquisition Date	2022-05-03T11:48:43
15 Modification Date	2022-05-03T11:48:44
16 Spectrometer Frequency	400.13
17 Spectral Width	8012.8
18 Lowest Frequency	-405.2
19 Nucleus	1H

^{1}H NMR (400 MHz, DMSO-d_6) δ 9.89 (s, 2H), 8.65 (dd, J = 21.1, 9.1 Hz, 4H), 7.85 (d, J = 9.1 Hz, 2H), 7.41 – 7.05 (m, 4H).

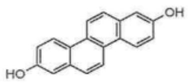

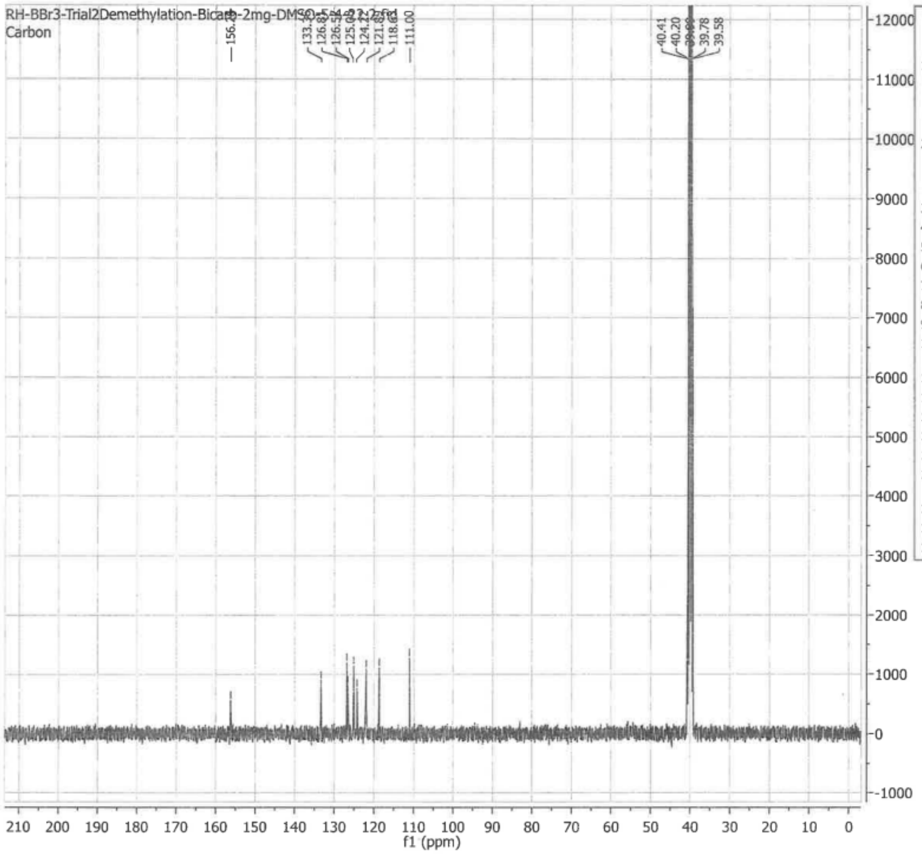

Parameters		
	Parameter	Value
1	Data File Name	C:/ Users/ Rama/ Desktop/ Rachel/ RH-BBr3-Trial2Demethylation-Bicarb-2mg-DMSO-5-4-22/ 2/ fid
2	Title	RH-BBr3-Trial2Demethylation-Bicarb-2mg-DMSO-5-4-22.2.fid
3	Comment	Carbon
4	Origin	Bruker BioSpin GmbH
5	Owner	nmrsu
6	Solvent	DMSO
7	Temperature	298.9
8	Pulse Sequence	zgpg30
9	Experiment	1D
10	Probe	Z108618_0813 (PA BBO 400S1 BBF-H-D-05 Z)
11	Number of Scans	12000
12	Pulse Width	14.8700
13	Acquisition Time	1.3631
14	Acquisition Date	2022-05-05T08:12:13
15	Modification Date	2022-05-05T08:12:13
16	Spectrometer Frequency	100.62
17	Spectral Width	24038.5
18	Lowest Frequency	-951.8
19	Nucleus	13C

^{13}C NMR (101 MHz, DMSO) δ 156.26, 133.36, 126.81, 126.57, 125.08, 124.22, 121.89, 118.63, 111.00, 40.41, 40.20, 39.99, 39.78, 39.58.

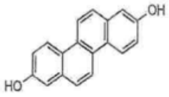

496

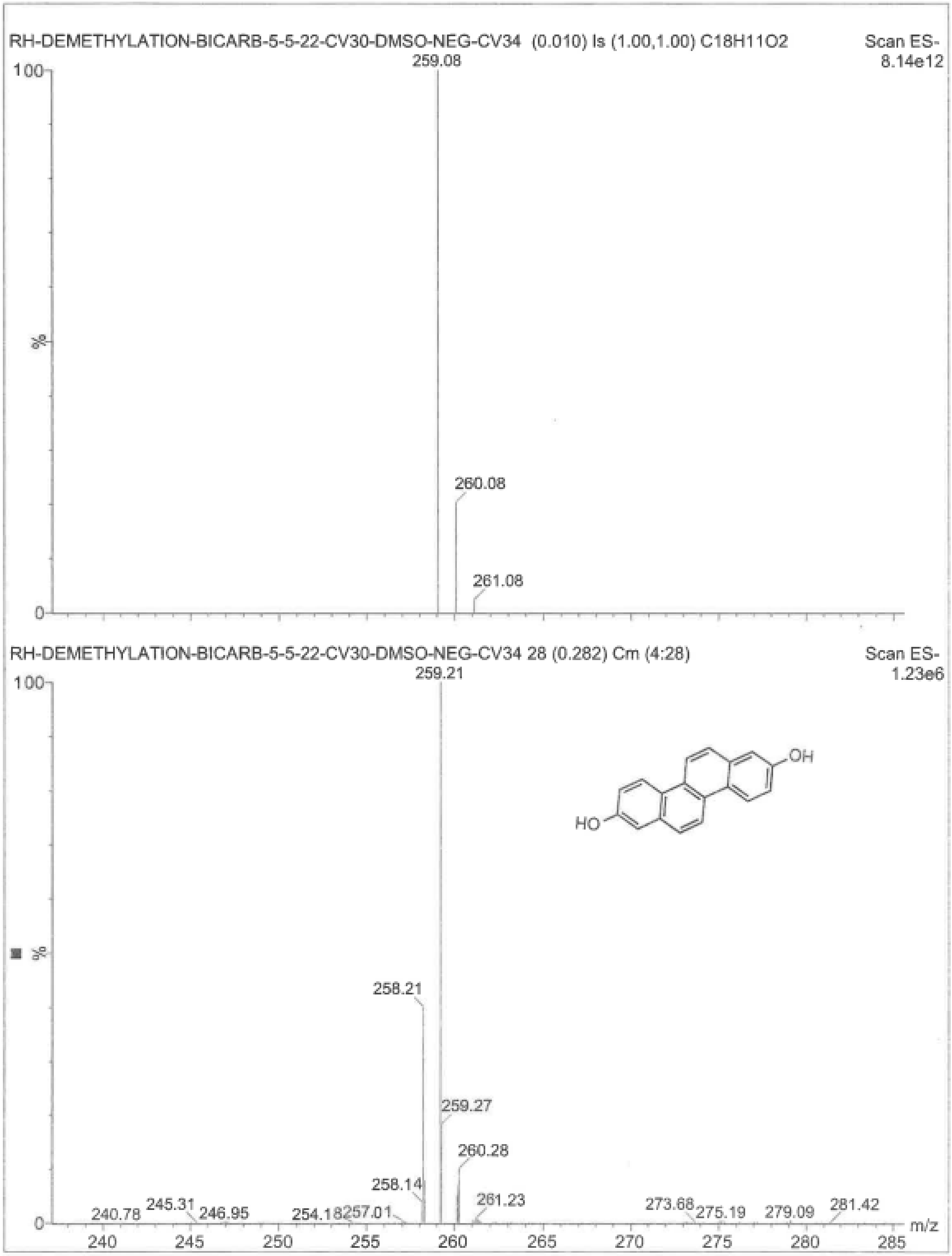

RH-DEMETHYLATION-BICARB-5-5-22-CV30-DMSO-NEG-CV34 (0.010) Is (1.00,1.00) C18H11O2
Scan ES-
8.14e12
259.08
260.08
261.08
100
%
0
RH-DEMETHYLATION-BICARB-5-5-22-CV30-DMSO-NEG-CV34 28 (0.282) Cm (4:28)
Scan ES-
1.23e6
259.21
258.21
259.27
260.28
258.14
261.23
240.78
245.31
246.95
254.18
257.01
273.68
275.19
279.09
281.42
OH
HO
m/z
240
245
250
255
260
265
270
275
280
285
